# Remote Marker-Based Tracking for UAV Landing Using Visible-Light Camera Sensor

**DOI:** 10.3390/s17091987

**Published:** 2017-08-30

**Authors:** Phong Ha Nguyen, Ki Wan Kim, Young Won Lee, Kang Ryoung Park

**Affiliations:** Division of Electronics and Electrical Engineering, Dongguk University, 30 Pildong-ro 1-gil, Jung-gu, Seoul 100-715, Korea; stormwindvn@dongguk.edu (P.H.N); yawara18@dongguk.edu (K.W.K); lyw941021@dongguk.edu (Y.W.L)

**Keywords:** unmanned aerial vehicle (UAV), remote marker-based tracking, visible light camera sensor, UAV landing

## Abstract

Unmanned aerial vehicles (UAVs), which are commonly known as drones, have proved to be useful not only on the battlefields where manned flight is considered too risky or difficult, but also in everyday life purposes such as surveillance, monitoring, rescue, unmanned cargo, aerial video, and photography. More advanced drones make use of global positioning system (GPS) receivers during the navigation and control loop which allows for smart GPS features of drone navigation. However, there are problems if the drones operate in heterogeneous areas with no GPS signal, so it is important to perform research into the development of UAVs with autonomous navigation and landing guidance using computer vision. In this research, we determined how to safely land a drone in the absence of GPS signals using our remote maker-based tracking algorithm based on the visible light camera sensor. The proposed method uses a unique marker designed as a tracking target during landing procedures. Experimental results show that our method significantly outperforms state-of-the-art object trackers in terms of both accuracy and processing time, and we perform test on an embedded system in various environments.

## 1. Introduction

The global market for unmanned aerial vehicles (UAVs) has grown dramatically in recent years along with rapid development of new applications [1]. UAVs haves been widely adopted in robotics research because they can extend human’s capabilities in a variety of areas especially for military application such as search-and-rescue and surveillance, as well as applications such as transportation, artistic photography and video. Typical UAVs are controlled by humans, and experience is required as the UAV still haves low control accuracy. The mission path of autonomous UAVs requires them to fly at low speed while following a path or to track an object of interest, and to perform a series of actions. Nevertheless, in order to address a broader range of applications, one has to migrate to integrated processing add-ons that would carry out on demand, on-board, collaborative or autonomous functions, with the aim of realizing an intelligent UAV functionality. With the increase in the computational potential of UAVs, previous studies do not only address computationally demanding tasks, but have adopted the UAVs into autonomous system that can take control of its own flight and perform optimized missions.

UAVs have exhibited performance in many challenging tasks that traditionally require significant human resource such as the inspection of infrastructure [2,3,4,5,6], indoor navigation using simultaneous localization and mapping [7,8,9], obstacle avoidance [10,11,12,13,14], terrain reconstruction [15,16], and real-time monitoring [17,18]. Drones can become a valuable tool to support humanitarian actions that aims to analyze characteristic of subject in order to determine the trends for future research directions [19]. In addition, they can be used to identifying and rescue potential victims [20]. Recently, Amazon, which is a giant E-commerce company successfully developed a delivery system designed to deliver packages to customers within 30 min using a UAV service called Amazon Prime Air [21]. It uses an Amazon-branded landing mat as a homing-beacon for the drone to land and deposit its payload. In general, however global positioning system (GPS) signal is usually owing to obstruction from tall building in urban environment.

Inspired by the amazing work from Amazon and considering the scenarios that result in GPS signal being lost, we propose a remote marker-based tracking algorithm for UAV landing procedure for various time and condition (during morning, afternoon, evening, and night time) without the need for any support from GPS signal. Our algorithm has two basic requirements, which are implemented on an onboard system: (1) real-time: the tracking algorithm can be processed on the onboard system at the real-time speed; and (2) accuracy: in order to successfully land on the landing pad, the tracking algorithm should track the marker precisely even in the presence of the aforementioned challenging factors.

The outline of the paper is organized as follows: In Section 2, we present recent works related to vision-based autonomous landing for drones. In Section 3, we introduce the proposed visual marker tracking algorithm. Then in Section 4, we discuss the performance evaluations in various conditions during a given day and we make comparisons with popular state-of-the-art visual trackers. Finally, we conclude the paper in Section 5.

## 2. Related Works

Previous studies of the vision-based landing for UAVs are classified into two categories, namely passive and active methods. Previous researches on passive methods utilized the camera sensors distributed on the ground and the complicated set up for both UAV or ground environments is necessary. Martínez et al. designed a trinocular system, which is composed of three FireWire cameras fixed on the ground, to estimate the vehicle’s position and orientation by tracking color land markers on the UAV [22]. Kong et al. deployed a custom-built infrared stereo camera with large field of view and claimed that their system could resist all weather conditions [23]. In his research, he successfully implemented several algorithms to track the UAV during landing operation. However, the accuracy is still low in case of fixed-wing touchdown points and high temperature objects in the background. Recent work from Yang et al. [24] shows the promising results on UAV auto landing in GPS-denied environment using a ground-based near infrared (NIR) camera system. Using an infrared laser lamp mounted at the nose of the UAV, their system achieved high landing accuracy with the distance over 1000 m. There is no denial that these passive methods achieved impressive results, but in some circumstances such as urban or low altitude operations, the issue for setting up a ground station with complicated equipment should be considered to be solved.

With respect to solving the landing problem in a much more convenient and less complicated way, active methods are considered, which use on-board camera mounted on the UAV to detect interested region or fiducial marker on the ground for accurate landing. They can be classified into marker-less and marker-based approaches. With respect to the former category, Anitha et al. [25] proposed a simple algorithm to estimate the relative position of the UAV and a runway in order to perform automatic landing based on the images captured by camera. While this algorithm performs well in the daytime, guiding lamps should be attached on both sides of the runway, making it difficult for use in various places. Using a single onboard camera, Li presents a two-stage processing procedure to find and evaluate all possible landing areas in order to select the best one for landing. To do so, they use machine learning algorithm based on naive Bayesian classifier [26]. However, this study did not perform the experiments using multiple images captured in various places and times, and the performance will be affected by the kinds of image.

To consider the limitation of the marker-less approach, several marker-based methods have been researched. Taking advantages of features pertaining to markers, recent studies on precise UAV landing have achieved improved landing performance. The marker center and direction are predicted for each input image in order to guide the drone to land at the marker’s center position with the correct orientation.

Sharp et al. used a visible light camera to capture an input image, and a square marker with white border and black background including a few smaller white squares inside, to simplify its segmentation from the background. They used corner detection and correspondence matching [27]. The circle patterned-marker has been used, and it could be identified from various heights by applying a fixed threshold to the input image followed by the contour detection to detect the concentric rings inside [28]. An improved version of speeded up robust features (SURF) was proposed by Zhao et al. [29] to resolve the inefficiency of the SURF algorithm in the autonomous landing system. Recently, many studies [30,31] used AprilTag [32] as a landing target owning to its high-contrast, and two-dimensional (2D) tags are designed to be robust to low image resolution, occlusions, rotations and lighting variation. Kyristsis et al. [33] used AprilTags C++ Library [34] along with the OpenCV4Tegra framework [35], which allows the performance of all OpenCV functions in parallel as graphics processing unit (GPU) functions and finally achieved the detection rate of 26–31 fps with the help of the global navigation satellite system (GNSS). The hardware that they used was quite powerful, and they employed a DJI Matrice UAV [36] along with an NVIDIA Tegra K1 SOC embedded processor [37]. These studies could successfully track the marker when the marker was clearly visible in the input image captured at daytime, but they would fail if the images become too dark at nighttime. To consider this issue, other studies show that infrared radiation images captured from a thermal imager may increase the rate of identification for the nighttime scenario [38,39]. The target emits far infrared (FIR) light actively in order to overcome the problem involving the incorrect detection of targets under low-light conditions. Using a letter-based marker, they can easily detect feature points so that the drone can perform translation or rotation movements in order to perform safe landing at the desired location. However, in these studies, expensive thermal cameras are required [38,39], and this is not possible in conventional drone system including only visible-light camera.

In our research, we focus on tracking the marker using one visible-light camera in a real-time manner on a common onboard system having a low processing power during both daytime and nighttime. Therefore, it is difficult to use high-computation algorithm [40,41,42,43] or features detection and description [44,45,46,47]. To do that, we propose two different tracking strategies to precisely land a UAV using a robust tracking algorithm regardless of whether the marker is visible during the day-time or not visible during the nighttime. Our research was novel compared to previous work in the following three ways:

(1) Our algorithm can track and detect a marker not only when the marker is visible in the morning, afternoon, and evening, but also when the marker is hardly visible at nighttime.
(2) Using images captured from a single visible-light camera with an onboard system that has low processing power, our algorithm outperforms previous state-of-the-art object trackers in terms of both accuracy and processing speed.
(3) Our marker design is simple and unique compared to those used by other marker-based tracking algorithm. Our database was self-constructed using a visible-light camera mounted on the DJI Phantom 4 drone [36] at various time during the day, and this database was made public so that other researchers can compare and evaluate its performance.

A comparison of previous tracking algorithms employed for autonomous drone landing with our proposed method is summarized in Table 1. 

## 3. Proposed Method

### 3.1. Overview of the Proposed Method

**Figure 1:**
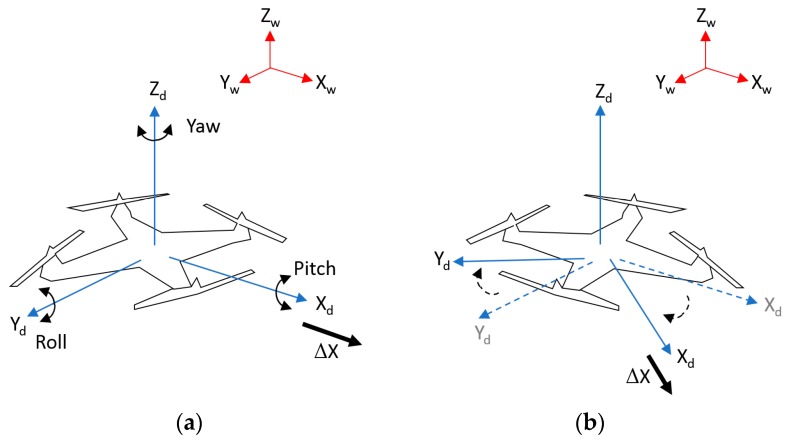
The two coordinates systems of drone and world (**a**) before changing the yaw of drone, (**b**) after changing the yaw of drone.

As mentioned in Section 1, our work focuses on supporting the safe landing of drone in sophisticated environments where the GPS signal is not available to use. Assume the scenario where the drone reaches the target destination with guidance from the GPS system, after which it begins to land to deliver its cargos or packages (delivery mission). Our objective is to solve the problem for the case when the GPS signal is not available, requiring the drone to depend on visual systems for landing operation. Our research does not deal with actual control or guidance of the drone while landing but we focus on implementing a high-speed vision-based tracking approach, enabling accurate landing at a desired location. In addition, using hardware drone stabilizer, the roll and pitch of the drone in Figure 1 can be maintained during the landing operation of drone. Therefore, we detect three parameters of translations for the X- and Y-axes (X_d_ and Y_d_ of Figure 1), and the yaw rotation of Figure 1, from which the drone can land at the correct position of the marker with the correct direction. The yaw (direction) estimation is required because the change of yaw rotation of the drone causes changes in the X- and Y-axes, which causes the incorrect translations of the drone. As shown in Figure 1a,b, even with the same translation of ΔX, the position of the drone varies by changing the yaw rotation based on global coordinate (X_W_, Y_W_, Z_W_).

In our research, we are not only trying to land the drone accurately during the daytime but also at nighttime. We used the information from the system clock of the embedded system and the image brightness level captured by drone camera to determine whether the operation is at day or night so that our algorithm would arbitrary select different tracking algorithms in the daytime (Section 3.2) and nighttime (Section 3.3).

### 3.2. Marker-Based Tracking Algorithm (Day Time)

#### 3.2.1. Proposed Marker Design

**Figure 2:**
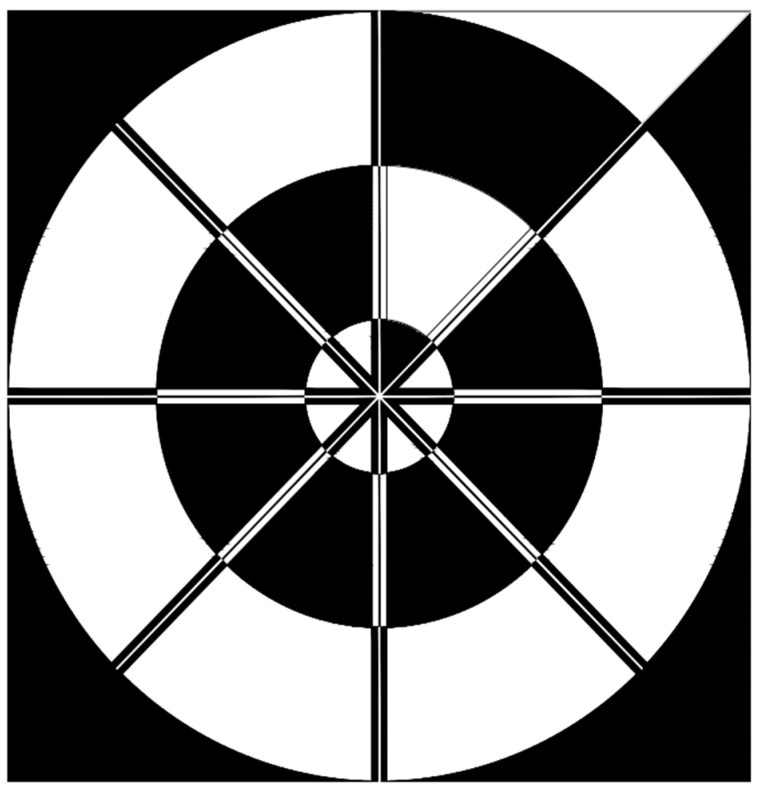
Our proposed design of marker for drone landing.

During the daytime, the marker is visible and if we can find 2D coordinates of the center of the marker, we can send a command to the drone to move closer to the center of the desired target. Our goal is to overcome the disadvantages of the commonly used patterns and create a target pattern that can be uniquely identified irrespective of the vertical distance Z from the drone to ground. In addition, the target pattern should be identified even when parts of the target are not visible. Moreover, the design pattern should be simple enough to be easily identified by vision algorithm at a high frame rate. Considering these requirements, our designed marker consists of three inner circles, each of which is evenly divided by 8 areas as shown in Figure 2. The width × height of our marker is 1 × 1 m. In order to make our marker unique and simple, we utilize an even distribution of black and white color in the area between the inner circles.

For example, in the area between the center of the marker and the smallest circle, there are seven white areas and one black area among the eight fan-shaped areas. The reason for putting one black area is that the direction of the drone should be maintained so as to move the drone based its axis, illustrated in Figure 1. The marker is printed on conventional fabric (polyester fabric) by MUTOH printer using water color ink [48]. The pixel contrast is 255 (0 for black area and 255 white region) in our marker image. Other detail specification including the reflectance factors of the paints is not open to the public because the commercial printer with ink was used for printing.

#### 3.2.2. Marker-Based Tracking Algorithm (During Day Time)

**Figure 3:**
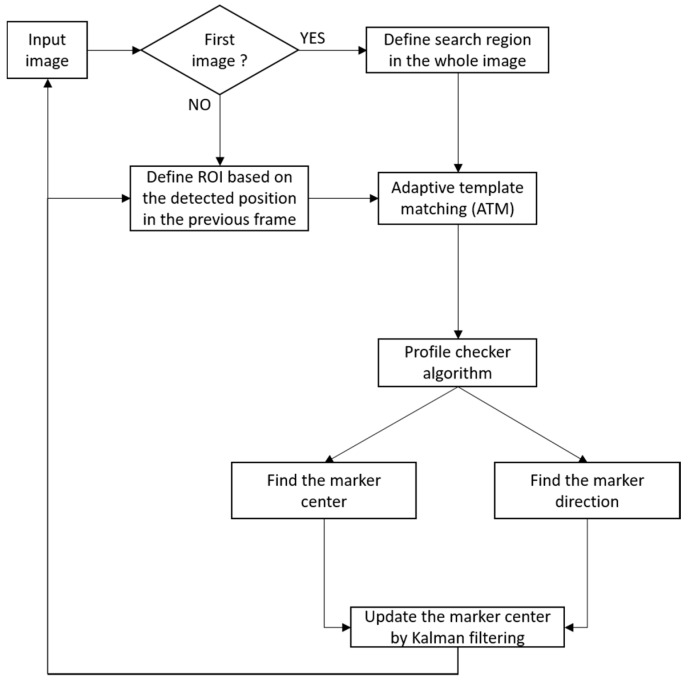
Flowchart of proposed marker-based tracking algorithm during daytime.

Figure 3 shows the overall flowchart of the proposed marker-based tracking algorithm during the daytime. Using the 1st image (captured by the camera) whose width and height are *W_o_* and *H_o_*, respectively, we reduce the size of the image by the factor of four (*W* = *W_o_*/4, *H* = *H_o_*/4) for faster processing in the next step of the template-matching process. There are several types of template matching, and we choose the correlation coefficient-based method because of its high matching accuracy [49]. Given *T* as the template image and *I* as a part of the input image, we obtained the normalized ones from both of them as *T’* and *I’*, and we used them for the correlation coefficient-based matching score *(M*) as follows.

(1) $$M=\frac{\sum T^{\prime }.I^{\prime }}{\sqrt{\sum {T^{\prime }}^{2}.{I^{\prime }}^{2}}}$$

In our research, our method is applied at the moment that GPS signal is lost. At this time, because the height of drone can be obtained based on the GPS signal, and the camera focal length with the marker dimension are known in advance, we can calculate the size of template. After that, because there is no additional information of height of drone including GPS signal, our method uses the same sized template. Using the template matching strategy, we obtained the center position of our marker, and we created a new template with the same size (*w* × *h*) as the original template. From the 2nd input image, we do not perform template matching for the whole image but in a small region of interest (ROI), The size of the ROI is (*w* + *m*) × (*h* + *m*) where *m* is a margin that we empirically considered large enough to ensure that the marker appears inside the selected ROI. We called this algorithm the adaptive template matching (ATM) algorithm because in every frame, the template image is updated, after which the change of the input image due to the drone landing and movement can be covered. However, there is drifting phenomenon of the detected center owning by the ATM algorithm based on the ground-truth center in each frame. Many previous studies [50,51,52,53,54,55] used template matching which has shown a large drifting phenomenon that significantly affects the tracking performance over long term. The reason for this is that during landing as the marker becomes bigger, its appearance changes rapidly, so the new (updated) template cannot keep up and therefore, in the long run, the tracking results deteriorate.

**Figure 4:**
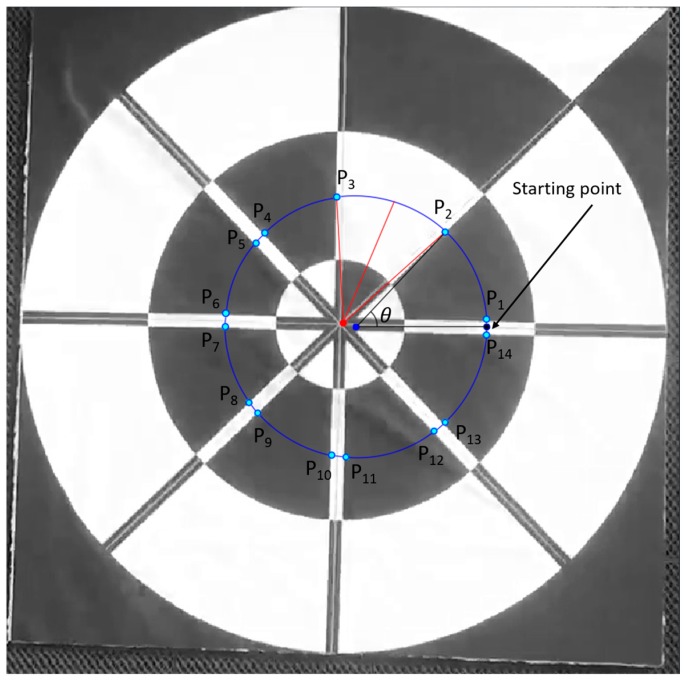
Predicted center and direction using our proposed method. Dark blue and red points respectively represent the positions detected by ATM and profile checker algorithm.

**Figure 5:**
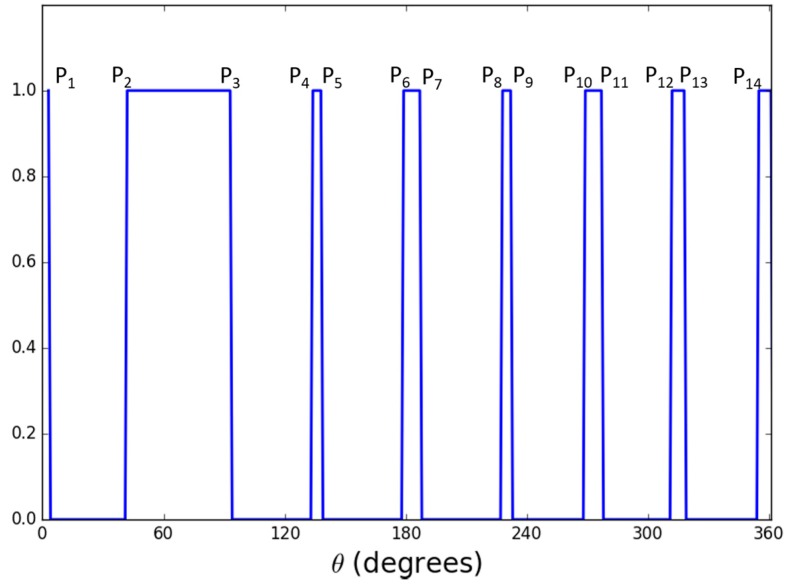
Binarized profile extracted from the measured circle of Figure 4.

In order to overcome this problem, we perform the profile-checker algorithm as shown in Figure 3. In our design of the marker of Figure 2, a black area is always between two white areas (in the circular direction) as shown in Figure 4. Therefore, based on the center of the marker, we can draw a circle that will contains 14 circular segments as shown in Figure 4 and Figure 5. We refer this circle as the profile and all of its segments as sub-profiles. Therefore, using the center detected by the ATM algorithm, as the angle *θ* increases from 0^°^ to 360^°^ as shown in Figure 4 and Figure 5, we obtain the values of all pixels along the circle (profile) in the counter-clockwise direction. With these values, we apply a threshold to create a profile with seven black and seven white sub-profiles (Figure 5). The threshold is defined as the mean value of the maximum and minimum value of all the obtained values. The value that is larger than the threshold is determined as 1; otherwise it is set to 0. From Figure 5, we observe that all black sub-profiles have a similar width while there is a white sub-profile that is wider than other white profiles. 

**Figure 6:**
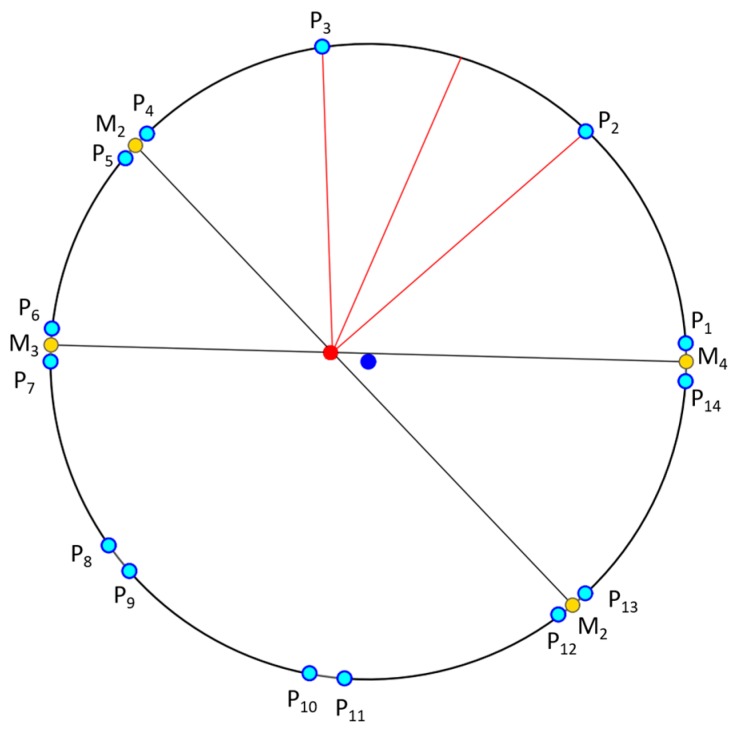
Our profile checker algorithm used to find the accurate center of the marker. Dark blue point is the incorrect center detected by the ATM algorithm, and the red point is the correct one detected by the profile checker algorithm.

Therefore, our proposed profile checker algorithm can detect the positions of P_1_, …, P_14_. In addition, we can detect M_1_, …, M_4_. Here, M_1_ is the midpoint between P_4_ and P_5_ as shown in Figure 6, and as in this method, we can also obtain M_2_, …, M_4_. Because the width of sub-profile P2–P3 is wider than those of others, we can differentiate this sub-profile from others. From the two points of $M_{1}$ and $M_{2}$, we obtained the line $\bar{M_{1}M_{2}}$. In addition, from those of $M_{3}$ and $M_{4}$, we obtained the line $\bar{M_{3}M_{4}}$ as shown in Figure 6. The intersect of these two lines is determined to be the marker center using the profile checker algorithm.

Our proposed profile checker algorithm can be described in detail as follows.

**Figure 7:**
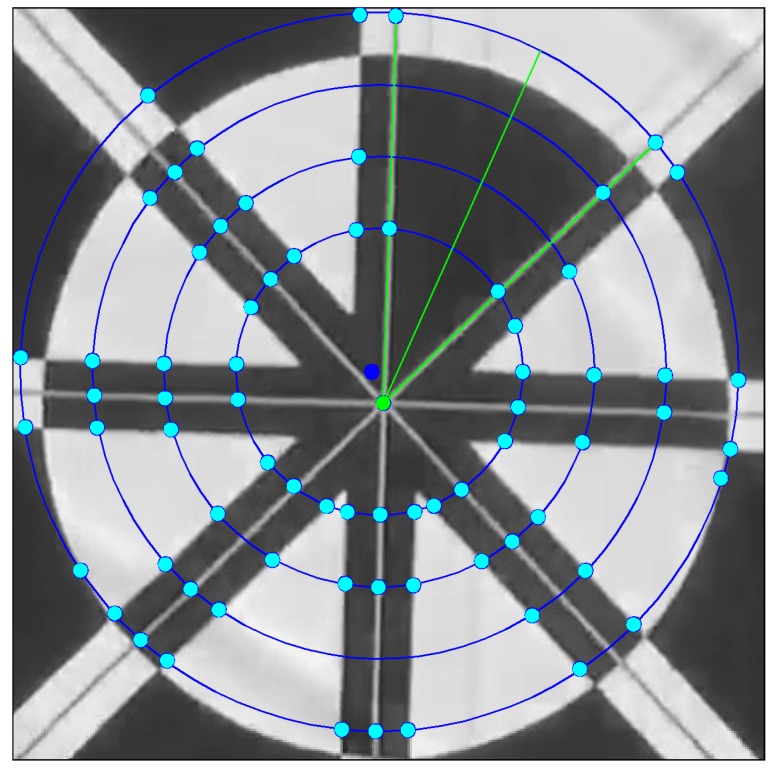
Strategy to predict marker center when the drone is close to the marker. Using the detected center (dark blue point) obtained by the ATM algorithm as the input for the Kalman Filter in the case where the profile checker algorithm fails, we obtained the corrected one (green point) as the final prediction. The direction of the marker is again estimated using the profile checker algorithm with the final center.

**Step 1**: Extract all pixel value in the circle profile with radius *r* in a vector: *V* = {*v*_1_, *v*_2_, …, *v*_360_}
**Step 2**: Find threshold *Th*: $Th=\frac{\max(V)+\min(V)}{2}$
**Step 3**: Obtain the binarized vector *V*′ = {*v_i_*′}_*i* = 1, …, 360_ where ${v_{i}}^{\prime }=\{\begin{array}{l} 0,{v_{i}}^{\prime }<Th\\ 1,{v_{i}}^{\prime }\geq Th \end{array}$
**Step 4**: Count the number of values (*C*) that are changed compared with previous value (from 0 to 1 or 1 to 0) and create *C* sub-profiles SP = {$\alpha_{k},\beta_{k},\gamma_{k},\delta_{k}$}_*k*= 1, …, *C*_, where $\alpha_{k},\beta_{k},\gamma_{k},\delta_{k}$ are the index of the starting point, index of the ending point, code (0 for black, 1 for white), and the width of the sub-profile (distance between the starting and ending point of the sub-profile), respectively
**Step 5**: If *C* is equal to 14, the sub-profile that has $\max(\delta_{k})$ is selected. Then, from the four adjacent sub-profiles of this selected sub-profile, two lines are detected (shown in Figure 6), and the intersect point of these two lines is determined as the **marker center by the profile checker algorithm**. Then, **the direction** is estimated based on the average position (between the starting and ending index of the selected sub-profile) and this detected center.
**Step 6**: If *C* is not equal to 14, we increase the radius of the circle profile of $R=r_{o}\pm i.\Delta r$ (where $\Delta r=\frac{w}{8},r_{o}=\frac{w}{4}$, and *w* is the width of template) as shown in Figure 7, and **steps 1~5** are repeated until $R<\frac{w}{2}$ (or *C* is equal to 14). If $R\geq \frac{w}{2}$, we use the detection result obtained by ATM as the marker center.

#### 3.2.3. Updating the Detected Center of Marker by Kalman Filtering

Using our proposed profile checker algorithm, we find the accurate center position and direction of the marker. However, the detected center position can still can vibrate based on the ground-truth center by the rapid change in the input image, and to solve this problem, we use Kalman filtering. Kalman filtering is a framework for predicting a process’s state and the use of new measurements to correct or update these predictions. For each time step *k,* a Kalman filter first makes a prediction ${\hat{x}}_{k}^{-}$ of the state at this time step:

(2) $${\hat{x}}_{k}^{-}=Ax_{k-1}+Bu_{k}$$

where $x_{k-1}$ is a vector that represent the processing time at state *k* − 1 and *A* is the process transition matrix. $u_{k}$ is a control vector at time step *k* and *B* converts the control vector $u_{k}$ into state space [56]. In our model of moving marker on 2D camera images, state is a 4 dimensional-vector ${\left[x,y,dx,dy\right]}^{T}$ where *x* and *y* represent the X- and Y-coordinates of the marker’s center, respectively. *dx* and *dy* represent its velocity on X- and Y-axes, respectively. For simplicity, we choose to use the following transition matrix:

(3) $$A=\left[\begin{array}{cccc} 1 & 0 & 1 & 0\\ 0 & 1 & 0 & 1\\ 0 & 0 & 1 & 0\\ 0 & 0 & 0 & 1 \end{array}\right]$$

Our UAV is able to make yaw rotation based on marker direction’s prediction so that $u_{k}$ is just a scalar representing how much the object is expected to move along the X- and Y-axes in response to control. Converting $u_{k}$ into state space is simple by using the *B* vector of Equation (4).

(4) $$B={\left[\begin{array}{cccc} 1 & 1 & 0 & 0 \end{array}\right]}^{T}$$

The Kalman filter concludes the time update steps by projecting the estimated error covariance $P_{k}^{-}$ forward by one-time step:

(5) $$P_{k}^{-}=AP_{k-1}A^{T}+Q$$

where $P_{k-1}$ is a matrix that represent the error covariance having full diagonals in the state prediction at time *k*, and *Q* is the process noise covariance. In our research, we choose a fixed process noise covariance as below:

(6) $$Q=\left[\begin{array}{cccc} 0.1 & 0 & 0 & 0\\ 0 & 0.1 & 0 & 0\\ 0 & 0 & 0.1 & 0\\ 0 & 0 & 0 & 0.1 \end{array}\right]$$

After the state $x_{k}^{-}$ is predicted at time *k*, the Kalman filter uses a new measurement to correct the prediction during the subsequent update steps. At the 1st input image, we initialize the Kalman filter and use the center detected by the profile checker algorithm as the measurements for the next step. Initially, we compute the Kalman gain and it is later used to correct the state estimate $x_{k}^{-}$:

(7) $$K_{k}=P_{k}^{-}H^{T}{\left(HP_{k}^{-}H^{T}+R_{k}\right)}^{-1}$$

where *H* is the matrix that convert the state space into measurement space and $R_{k}$ is the measurement noise covariance. In our case, we use a fixed $R_{k}$ for all future time updates:

(8) $$R_{k}=\left[\begin{array}{cc} e^{-3} & 0\\ 0 & e^{-3} \end{array}\right]$$

where *e* is Euler’s number. Using the Kalman gain $K_{k}$ and measurement $z_{k}$ from time step k, we can update the new estimate:

(9) $${\hat{x}}_{k}={\hat{x}}_{k}^{-}+K_{k}(z_{k}-H{\hat{x}}_{k}^{-})$$

In our approach, measurements $z_{k}$ are the output from our proposed marker tracking algorithm so that $z_{k}$ contains two dimensions and has the form $\left[x_{0},y_{0}\right]$^T^. As a result, *H* has the form:

(10) $$H=\left[\begin{array}{cccc} 1 & 0 & 0 & 0\\ 0 & 1 & 0 & 0 \end{array}\right]$$

The final step of the Kalman filter at each iteration is to update the error covariance $P_{k}^{-}$ into $P_{k}$:

(11) $$P_{k}=(I-K_{k}H)P_{k}^{-}$$

However, in the case when the drone gets very closer to the marker like, as in Figure 7, even though we tried to increase the radius of the circle many times, the number of obtained sub-profiles is larger than 14 at the largest blue circle. In this case, we choose the center detected by ATM as the new measurement (dark blue point) for the Kalman filter. Using the final center detected by Kalman filtering (green point), we again perform the proposed profile checker algorithm and selected the sub-profile that has the largest width to find its bisecting angle as the final direction of the marker.

### 3.3. Marker-Based Tracking Algorithm (Night Time)

**Figure 8:**
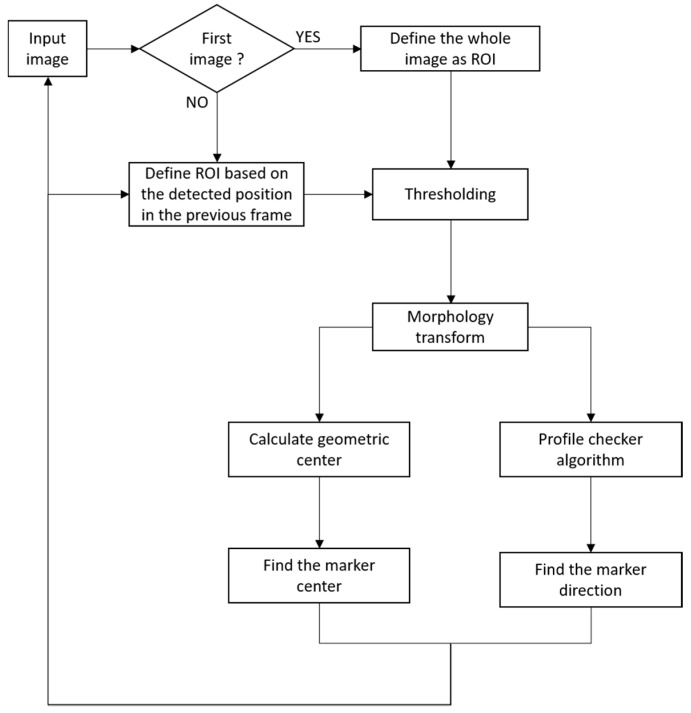
Flowchart of proposed marker-based tracking algorithm during night time.

**Figure 9:**
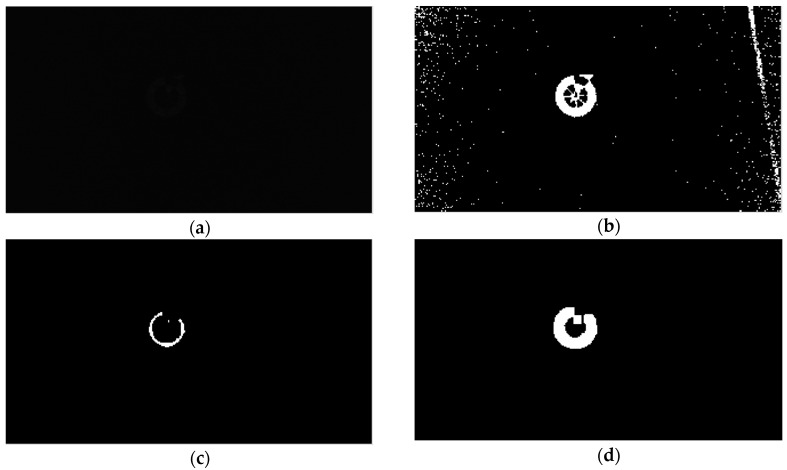
Our proposed marker segmentation results: (**a**) original image; (**b**) after applying adaptive thresholding; (**c**) after applying Hit and Miss morphology (**d**) final result after dilation.

In our research, we aim to develop an algorithm that works well in any kind of lighting conditions. Our strategy in the daytime is to take advantages of the unique design marker and to track its position as the drone’s altitude decreases. However, because the image is too dark at nighttime as shown in Figure 9a, the use of the same approach leads to errors in the marker detection because the ATM algorithm performs poorly when there is a very small difference between the marker and non-marker areas. As explained in Section 3.1, we use the information obtained from the system clock of the embedded system and the image brightness level captured by drone camera to determine whether the operation is during the daytime or nighttime. Therefore, our algorithm would arbitrary select different tracking algorithms base on the time of the day. In detail, our algorithm does not depend on only the time (the system clock of drone), but refers to both the time and image brightness level captured by the drone camera. The reason why our method does not depend on only the image bright level is that it can be affected by the brightness of background such as bright or dark ground. Therefore, our algorithm first checks the time. If the time is at night, our method checks the image brightness level again for the higher credibility of determination of daytime and nighttime. If the image brightness level is lower than threshold, then our method performs the tracking algorithm for nighttime of Figure 8. If either the time or image brightness level is not satisfied with our condition, our method determines that it is daytime and performs the tracking algorithm for day time of Figure 3.

Figure 8 shows the overall marker detection procedure at nighttime. At nighttime, image segmentation is performed to roughly estimate the position of the marker from the input image. Of the various image segmentation techniques [57,58,59,60,61], we propose a simple segmentation algorithm to distinguish our marker and the background from the input image. First, we apply adaptive thresholding to the input image. The adaptive threshold is determined based on the brightness histogram of the ROI of the image, as well as the image-binarization algorithm [62]. There is also a need for noise to be removed in the image after thresholding. To do this, we used a morphology technique called the Hit and Miss algorithm [63]. The purpose of the Hit-and-Miss algorithm is to detect certain patterns in an image. A structure element containing 0, 1 or blank is used as a template that slides over the image and the pixel corresponding to the center of the template is set to 1 if the template matches the images or 0 otherwise. After that, a thin pattern of our marker is successfully detected as shown in Figure 9c; next we need to roughly estimate the area that belongs to the marker. To do that, we process the result using the dilation algorithm [63], which helps to increase the boundary to the background, as shown in Figure 9d.

**Figure 10:**
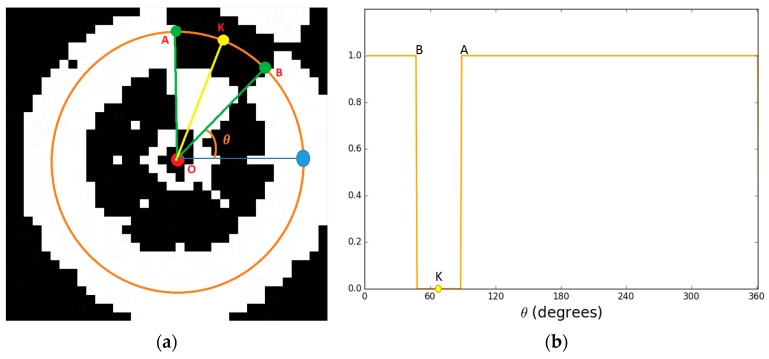
Detected center and direction of marker in (**a**) result image using our proposed method. (**b**) Profile visualization from the circle of Figure 10a.

The detected marker is displayed as white pixels while black pixels indicate the background area as shown in Figure 9d. We roughly estimate the center of the marker by calculating the geometric center of the white pixels. Based on that, we can obtain the width and height of the. Then, we apply our proposed profile checker algorithm as we try to find the direction available in the marker at nighttime. Using the result image (Figure 10a), we created a profile based on the predicted center. We empirically choose the radius of the circle profile as 0.4 × (width of marker). From the generated profile (Figure 10a), we see that there are only two sub-profiles: one black and one white. From that, we detect the “A” and “B” position in Figure 10b, and the direction is estimated based on the midpoint (“K”) of the arc connecting “A” and “B” with the detected center “O” of Figure 10a.

## 4. Experimental Results 

### 4.1. Experimental Platform and Environments

**Figure 11:**
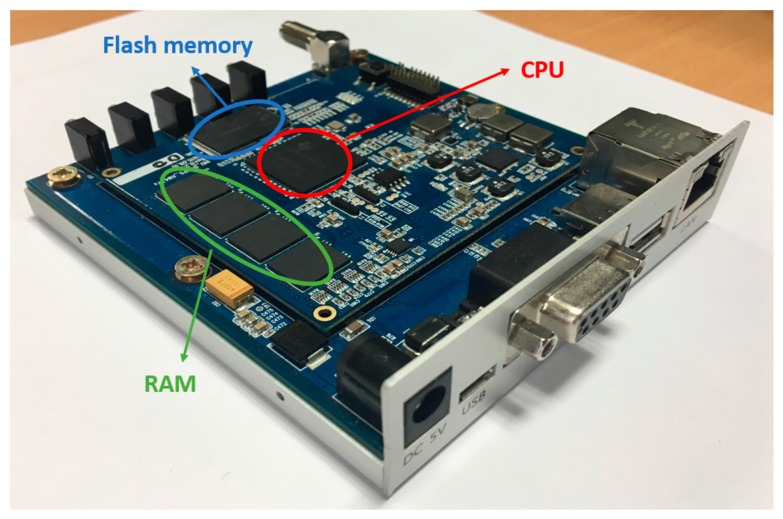
Our custom built embedded system.

In our experiments, we used a DJI Phantom 4 quadcopter [36] to capture the video while the drone was landing. It includes a color camera with a 1/2.3-inch-thick complementary metal–oxide–semiconductor (CMOS) sensor, with a 94° field-of-view (FOV) and an f/2.8 lens. The captured videos are in mpeg-4 (MP4) format with 30 fps, and have a size of 1280 × 720 pixels. For fast processing, in our experiment, the captured color image is converted to a gray one. By averaging the R, G, and B pixel values, we obtained the gray information. The drone’s gimbal is adjusted 90° downward so that during landing, the camera can be facing the ground. We implemented our algorithm on an onboard system that has a 32-bit 800-MHz ARM Cortex-A9 central processing unit (CPU) [64], 512 MB RAM, 1.5 GB flash memory, and a Linux kernel (version 3.12.10). In previous studies [33,65,66], they used a sophisticated tracking algorithm with a high-end embedded system such as an NVIDIA Jetson TK1 developer kit, including an ARM Cortex-A15 CPU (higher than 1 GHz [64]) and GPU [37], or an Intel NUC board with a 3.4 GHz CPU [67]. In particular, in [33,66], parallel processing is possible using a GPU, but our system does not include a GPU, which makes it difficult to utilize parallel processing. We developed our algorithm using an OpenCV library (version 3.1 [68]) and C++ program using Microsoft Visual Studio 2015 [69]. Then, we ported our program on the onboard system on which our system actually operates. Originally, our onboard computer did not support OpenCV library, and we created a custom OpenCV library using the ARM cross compiler tool and CMake software [70]. Then, we transferred it into our onboard system. Our onboard system is shown in Figure 11.

There are open databases that are captured by drone cameras, such as the Stanford Drone Dataset [71], Mini-drone Video Dataset [72], and SenseFly Dataset [73]. However, there are no open databases with images acquired while drones perform landing operations. Therefore, we acquired videos to build a new database (Dongguk Drone Camera Database (DDroneC-DB1) [74]) for our method. Our database (shown in Table 2) is divided in two sub databases: drone landing on the marker and drone hovering over the same position while the marker is moving on the ground. For each sub database, we captured four videos at 10 AM, 2 PM, 6 PM, and 10 PM. We acquired videos in varying types of environments (humidity level, wind velocity, temperature, and weather). The marker was visible in the sequences for the morning, afternoon, and evening, but it was barely seen in the night video. We made our DDroneC-DB1 public to other researchers through [74] to enable them to evaluate the performance of their marker-tracking methods using our database.

### 4.2. Experimental Results

#### 4.2.1. Marker Detection Accuracy and Processing Time

Using DDroneC-DB1, we compared the accuracies and processing time of our method with those obtained by the state-of-the-art methods of object tracking such as Multiple Instance Learning (MIL) [40], Tracking-Learning-Detection (TLD) [41], Median Flow [42], and Kernelized Correlation Filter (KCF) [43]. 

In this work, we calculated the center location error (CLE) and predicted direction error (PDE) of the tracked marker to compare the accuracies as follows:

(12) $$\mathrm{CLE}=\|O_{K}^{E}-O_{K}^{GT}\|$$

(13) $$\mathrm{PDE}=\|D_{K}^{E}-D_{K}^{GT}\|$$

where $O_{K}^{E}$ and $O_{K}^{GT}$ are the estimated and ground truth positions of the marker’s center, respectively. $D_{K}^{E}$ and $D_{K}^{GT}$ are the predicted and ground truth direction of the marker, respectively. In Table 3 and Table 4, we compared the CLE and the PDE obtained using our method with those obtained using other methods. The previous methods, i.e., MIL, TLD, Median Flow, and KCF do not produce the marker direction, but only the center of the marker. Therefore, we compared the PDE obtained by our method with those obtained by ATM algorithm, and with our method without Kalman filtering as shown in Table 4.

In our research, our goal is to find the marker in the current frame, which we have tracked successfully in previous frames. Conventional state-of-the-art trackers [40,41,42,43] use a bounding box manually provided at the first frame or by other detection algorithms, and they take it as the positive example for the object. Many image patches outside the bounding box are considered as the background. In order to test these trackers with our self-constructed dataset DDroneC-DB1, we perform template matching at the first image to select the initial bounding box which contains the marker and use it as the input of these state-of-the-art trackers. MIL tracker considered a small number of neighborhood locations around the predicted bounding box from previous step as positive examples and group them in a positive bag [40]. The collection of images in the positive bag are not all positive examples. Instead, only one image in the positive bag needs to be a positive example. Even if the current location of the tracked object is not accurate, when samples from the neighborhood of the current location are put in the positive bag, there is a good chance that this bag contains at least one image in which the object is nicely centered. TLD algorithm decomposes the long-term tracking task into three components: tracking, learning and detection [41]. The TLD tracker follows the object from frame to frame. The detector localizes all appearances that have been observed so far and corrects the tracker if necessary. The learning component estimates detector’s errors and updates it to avoid these errors in the future. In the other hands, Median Flow tracks the object in both forward and backward directions in time and measures the discrepancies between these two trajectories [42]. Minimizing forward and backward error enables them to reliably detect tracking failures and select reliable trajectories in video sequences. Recent KCF tracker builds on the idea of MIL tracker [43]. This tracker utilizes the fact that the multiple positive sample used in the MIL tracker have large overlapping regions. This overlapping data leads to some nice mathematical properties that is exploited by this tracker to make tracking faster and more accurate at the same time.

Although the state-of-the-art trackers [40,41,42,43] are designed to track generic objects, in our experiments, we optimized these state-of-the-art trackers for our marker for fair comparison. Although the performance of these trackers is lower than that by our method, the performance degradation of these trackers with the sub-database 2 (drone hovering) is lower than that with the sub-database 1 (drone landing). That is because these trackers are designed to track the object whose size does not change much. However, our method can track the marker even in the case that the size of marker changes drastically. The reason why we used these state-of-the-art trackers for comparisons is that there is no open source of mark tracker which can be used for our marker. The previous algorithms for ArUco, AprilTags, and Alvar cannot be used for our specific marker, but they can be used for their own types of marker. Therefore, we used these state-of-the-art trackers for comparisons.

As shown in Table 3 and Table 4, our method outperforms the other methods as well as our method without Kalman filtering (“Ours without KF”) in case of the sequences of morning, afternoon, and evening. However, for the night sequence, our method without Kalman filtering shows higher accuracies than our method with Kalman filtering (“Ours with KF”). Because the visibility of the marker is degraded at nighttime as shown in Figure 9a, the detection accuracy of the marker center obtained by our method is degraded compared to those obtained by our method in daytime. This increases the fluctuation of the detected position of the marker center at nighttime, and consequently, the errors of the Kalman filtering also increases. Therefore, our proposed algorithm does not use Kalman filtering at nighttime as shown in Figure 8.

**Figure 12:**
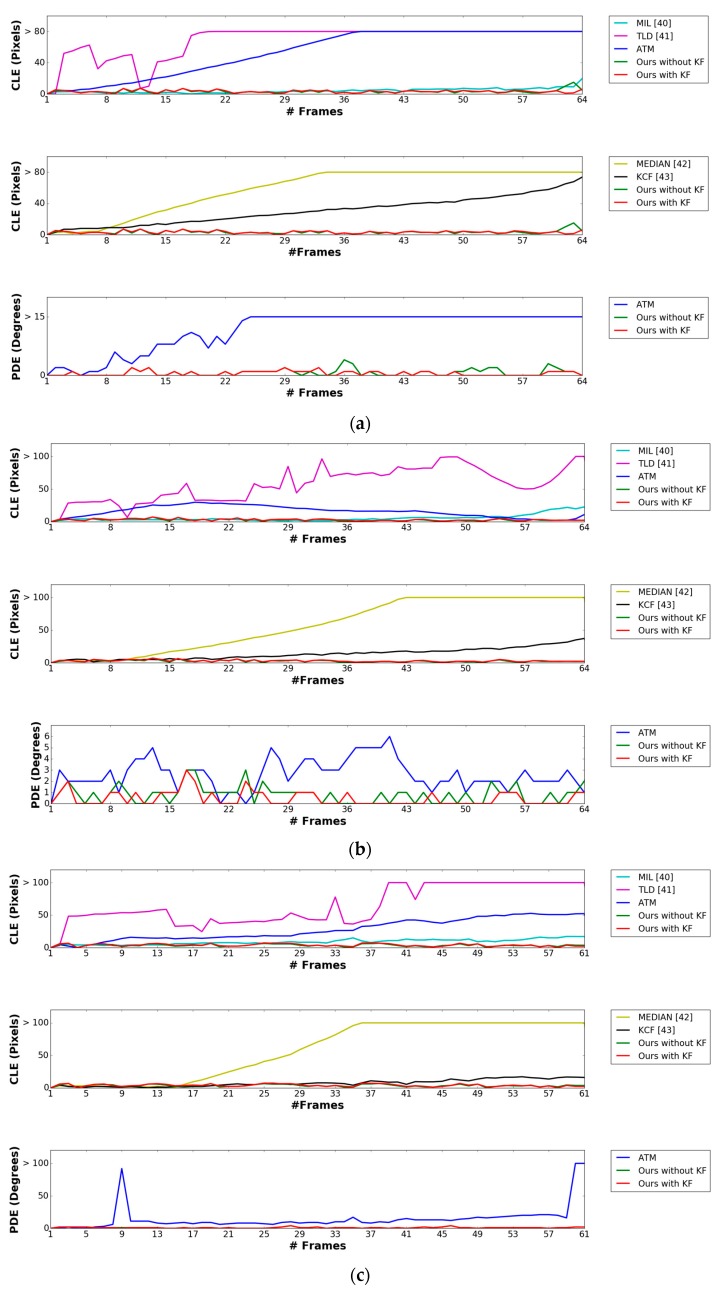

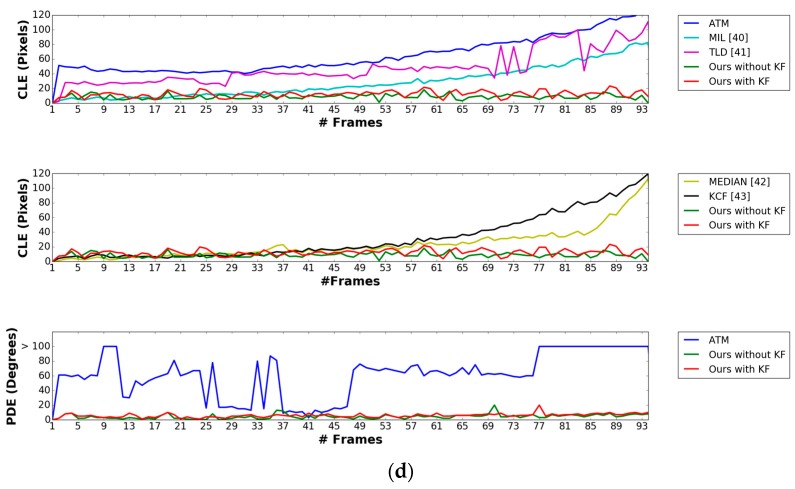
Comparisons of CLE and PDE obtained by our method with those obtained by other methods using sub-database 1 including the videos captured (**a**) in the morning, (**b**) in the afternoon, (**c**) in the evening, and (**d**) at night.

**Figure 13:**
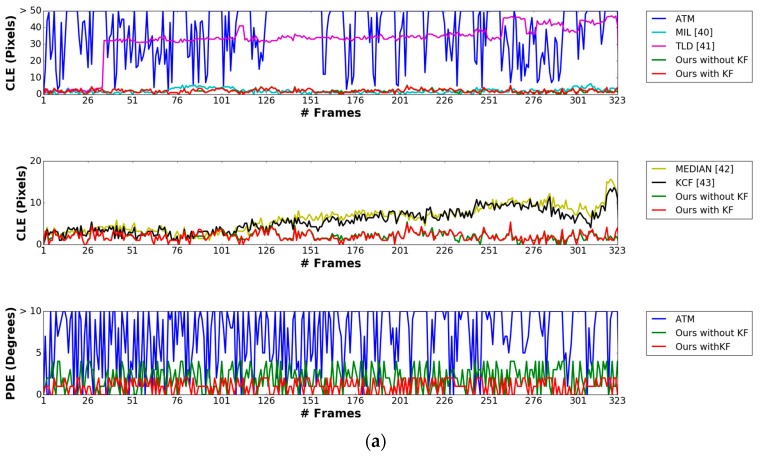

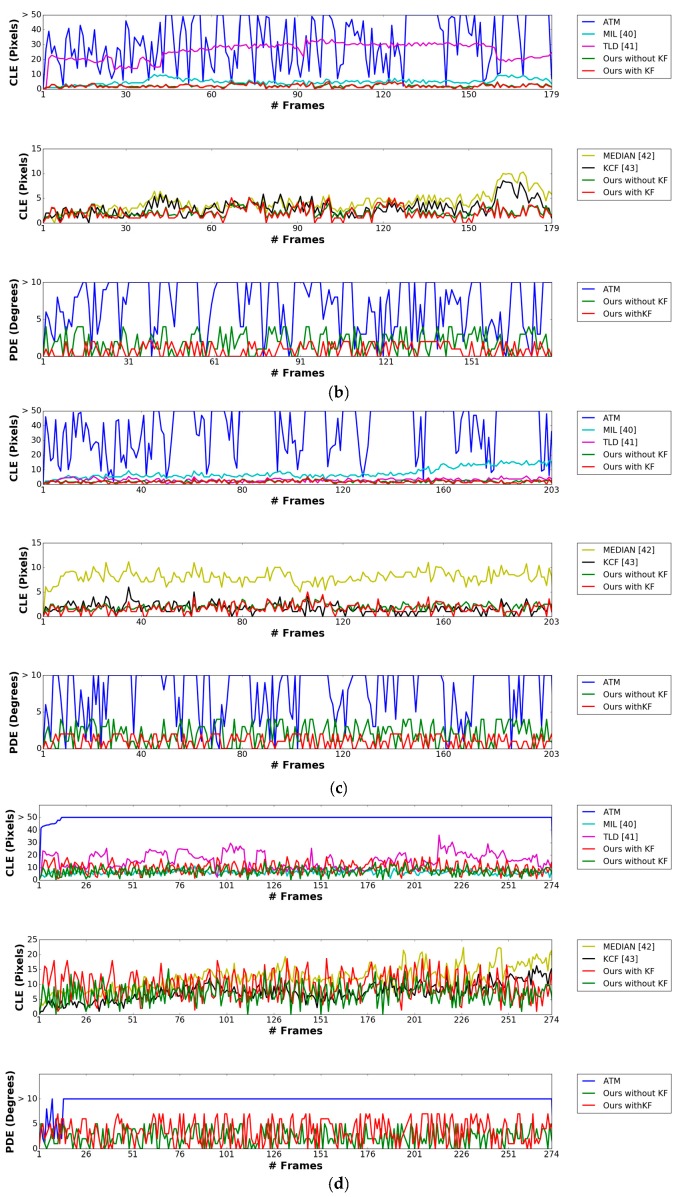
Comparisons of CLE and PDE obtained by our method with those obtained by other methods using sub-database 2 including the videos captured (**a**) in the morning, (**b**) in the afternoon, (**c**) in the evening, and (**d**) at night.

Figure 12a–d shows the comparative graphs of the CLE and PDE obtained by our method compared with previous methods using sub-database 1. Figure 13a–d shows those of the CLE and PDE with sub-database 2. As previously explained, the previous methods, i.e., MIL, TLD, Median Flow, and KCF do not produce the marker direction, but only the center of the marker. Therefore, we compared the PDE obtained by our method with those obtained by ATM, and our method without Kalman filtering (“Ours without KF” in Figure 12 and Figure 13). In addition, in Figure 12 and Figure 13, “Ours with KF” refers to our proposed method, and “MEDIAN” represents the method of Median Flow. As shown in Figure 12a–c, our proposed method outperforms the MIL, TLD, Median Flow, KCF, as well as our method without Kalman filtering in terms of CLE and PDE. In Figure 12d, although our method outperforms MIL, TLD, Median Flow, and KCF, our method without Kalman filtering shows higher accuracies than our method with Kalman filtering. That is because the visibility of the marker is severely degraded at nighttime as shown in Figure 9a, which causes the detection accuracy of the marker center obtained by our method to degrade compared to those obtained by our method in the daytime. This increases the fluctuation of the detected position of the marker center at nighttime, and consequently, the errors of the Kalman filtering also increases.

**Figure 14:**
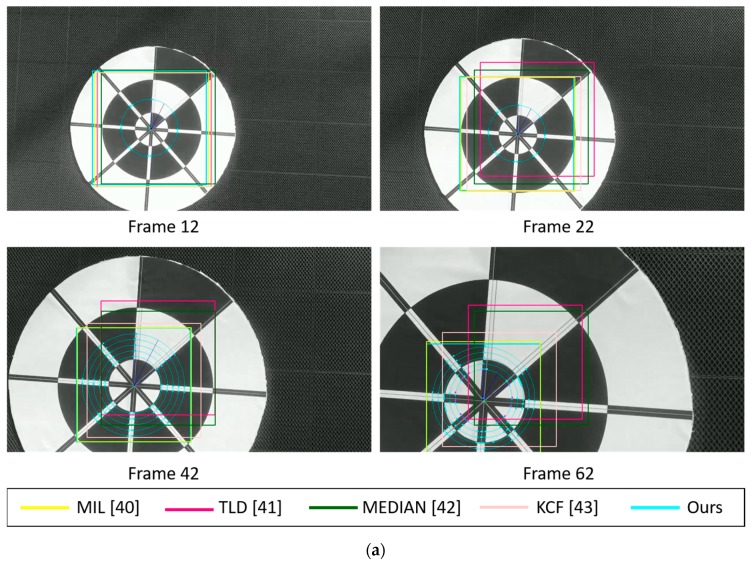

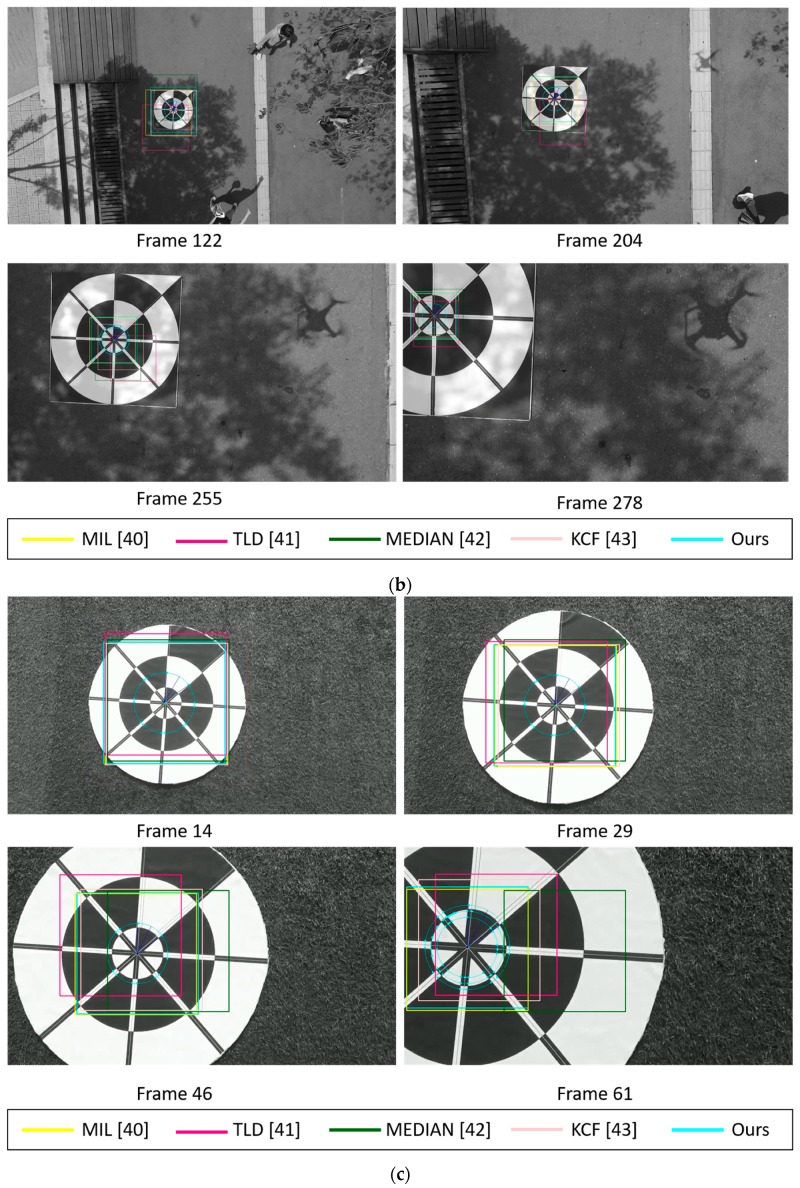

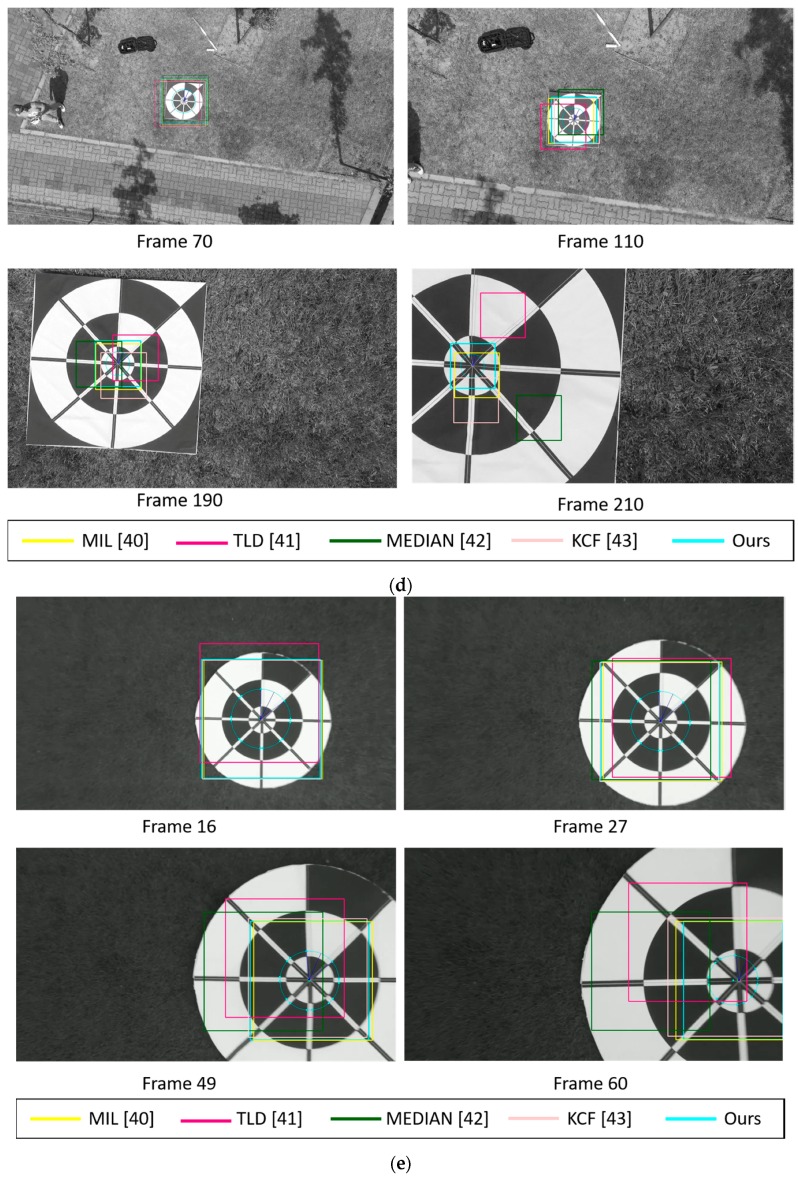

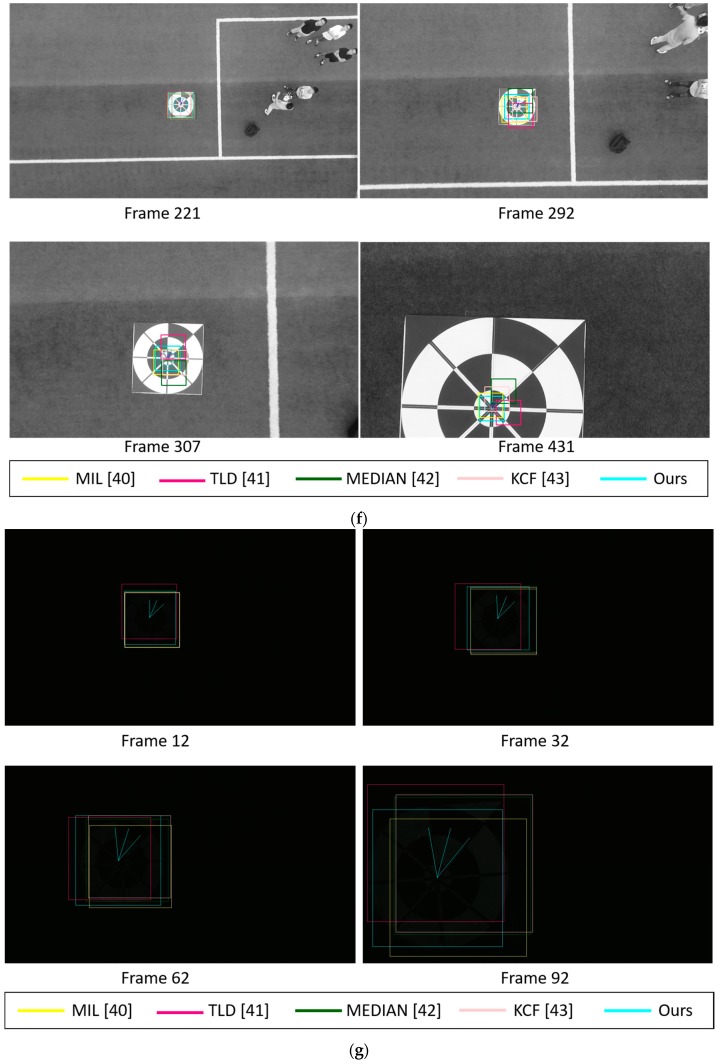
Marker detection examples obtained using our method and previous methods using sub-database 1 including videos captured (**a,b**) in the morning, (**c,d**) in the afternoon, (**e,f**) in the evening, and (**g**) at night.

Figure 14 shows the marker-detection examples obtained by our method and previous methods with sub-database 1. Our experiments conducted at two different heights: 6 m and 10 m in the case of morning, afternoon, and evening. For the night time case, we only test marker detection at the height of 6 m because images captured at the height further than 6 m are too dark and cannot perform any detection. In our experiments, we use the DJI Phantom 4’s remote controller in its default setting of Mode 2 (with the left stick controlling the throttle). At the heights of 6 m, we let the drone descend by manually pushing the left stick down until the drone safely lands on the ground and all the wings stop rotating. Meanwhile, at the height of 10 m, we let it descend using Return-to-Home (RTH) function.

As shown in Table 3 and Figure 14, MIL and KCF trackers are the 2nd and 3rd ranked in accuracy, respectively. Both of them have comparable results due to the similarity in the nature of their algorithms. We especially note that the TLD and Median Flow trackers easily lose target even when the marker is still in the FOV of the camera. In addition, our proposed marker tracker is able to locate the target accurately even in the case that part of marker disappears in the FOV of the camera. At the 62nd frame of Figure 14a, both TLD and Median Flow trackers completely lose the marker when some portions of the marker are out of view, i.e., some parts of the marker are not shown in the camera’s FOV. The same results can be observed at the 61st frame of Figure 14c and the 60th frame of Figure 14e. In the case of night landing, as shown in Figure 14g, our proposed method maintains a good bounding box prediction which covers the most area of the marker while other trackers fail.

In the 10 m landing experiments, scale variation and cluttered background are the main challenging factors. We compare marker detection at many different areas and environments. For example, as shown in Figure 14b,d, test images are both captured in a sunny day but at different environments. We purposely put our marker under the shade of a tree with lots of noisy lighting hole in Figure 14b in order to check our proposed method’s robustness. Meanwhile, in Figure 14d, our marker is placed at an open area which has a very strong sunlight. In contrast, our marker in Figure 14f looks very blurred at the height of 10 m. Despite having several challenges, our proposed tracker maintains higher accuracy compared to other trackers, followed by MIL, KCF, Median Flow and TLD trackers.

**Figure 15:**
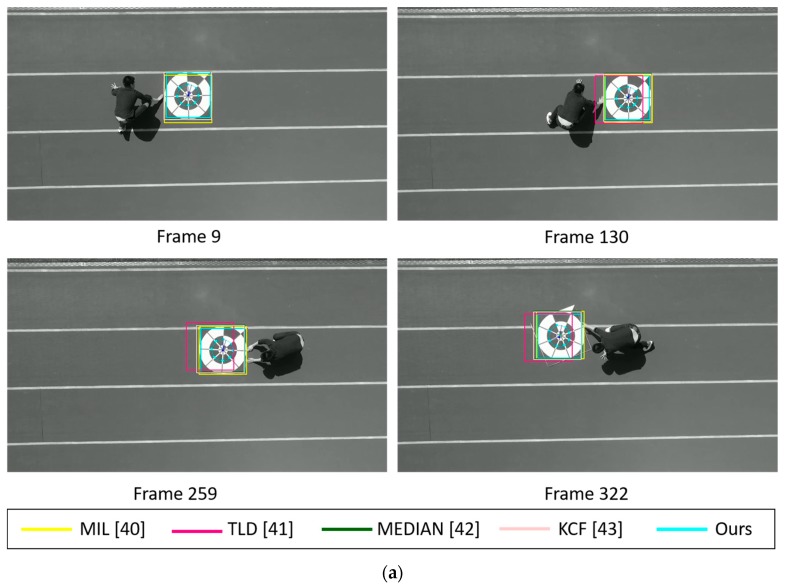

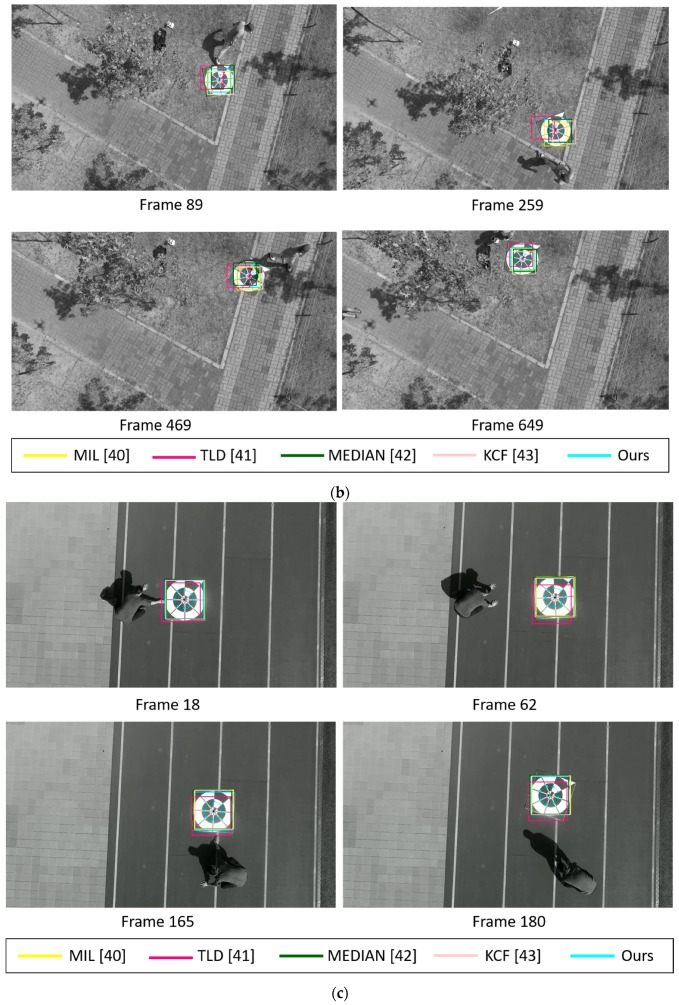

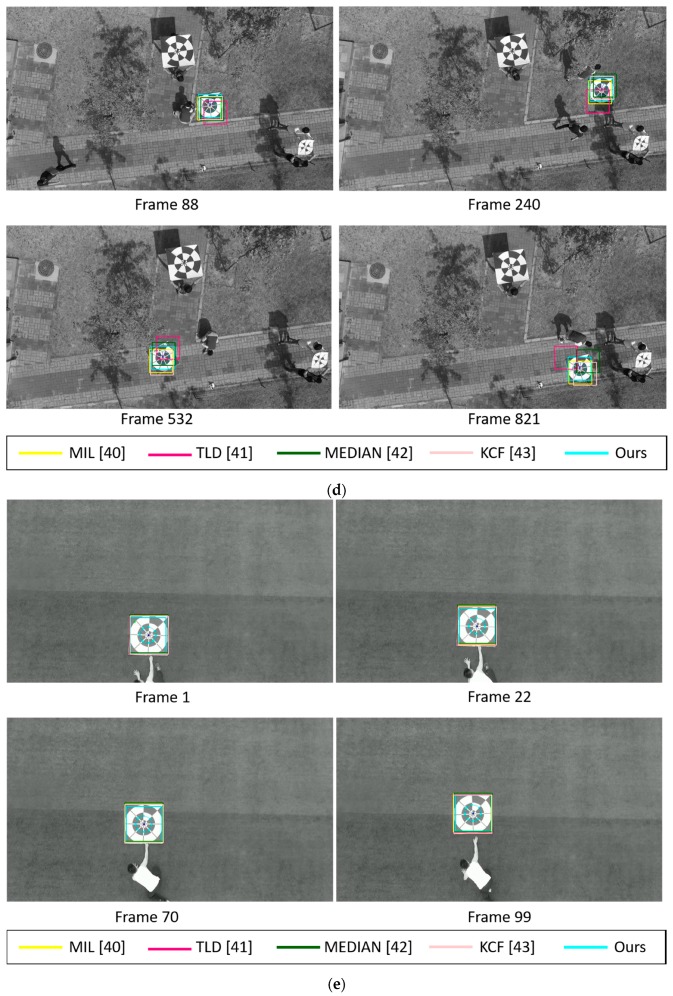

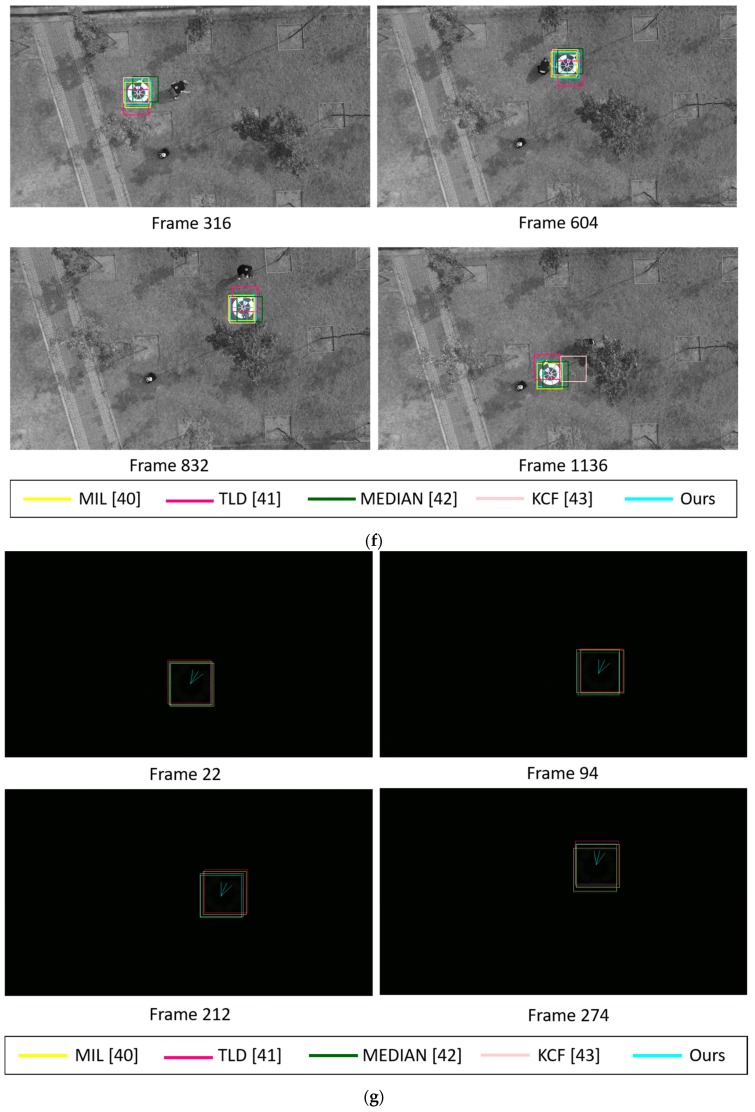
Marker detection examples by our method and previous methods using sub-database 2 including the videos captured (**a**,**b**) in the morning, (**c**,**d**) in the afternoon, (**e**,**f**) in the evening, and (**g**) at night.

In addition, Figure 15 shows the marker-detection examples by our method and previous methods with sub-database 2 at the height of 10 m. Different from the previous experiments, we tested our algorithm and previous methods to determine the detection results while the marker is translated or rotated on the ground. This type of experiment is similar with other state-of-the-art’s general object tracking test. As shown in Figure 15a,c,e, even though we manually translate marker with some little changes of orientation, our tracker has no problem detecting marker center and its direction in successive frames. A much more complex background with marker’s high-velocity translation and 360° rotations are main challenges in Figure 15b,d,f. Instead of slowly moving the marker by hands, we kick and push the marker further away which results in massive movements and rotations. Moreover, in Figure 15d, we design and print two toy markers with a similar design with our proposed marker and put them near our proposed marker in the testing scene. Our proposed tracking algorithm still achieves the best tracking performance and returns no false positive detection. We observe that there is no huge degradation of tracking performance from TLD and Median Flow trackers compared to previous landing test. MIL and KCF trackers successfully follow marker location and have reasonable performance. Overall, our method outperforms previous marker-detection methods, and our method can correctly detect the marker even in complicated backgrounds. In addition, even with the night images, our proposed method can detect both the correct position and direction of the marker, as shown in Figure 14g and Figure 15g.

Table 5 shows the comparative processing time per image obtained by our method and previous methods. We measured the processing time using the embedded system of Figure 11. As shown in Table 5, the processing speed achieved our method is much faster than those achieved by previous methods, and our method can be operated at a real-time speed of more than 40 (1000/25) fps. Although MIL shows a lower marker detection error than our method with the morning and night videos of sub-database 2, as shown in Table 3, the processing speed of MIL is too slow for use in real-time embedded systems compared to our method as shown in Table 5. Therefore, the effectiveness of our method is higher than previous developed methods.

#### 4.2.2. Pose Estimation Experiments

In the next experiment, we compare the accuracy of full pose estimation by our method with that by fiducial marker tracker of ArUco. In order to compute the full pose of our detected marker with respect to the camera frame, we need to obtain following information: intrinsic parameters of the camera (camera matrix and distortion coefficients), 2D coordinates of a few points in the input image and their 3D locations in the real world.

**Figure 16:**
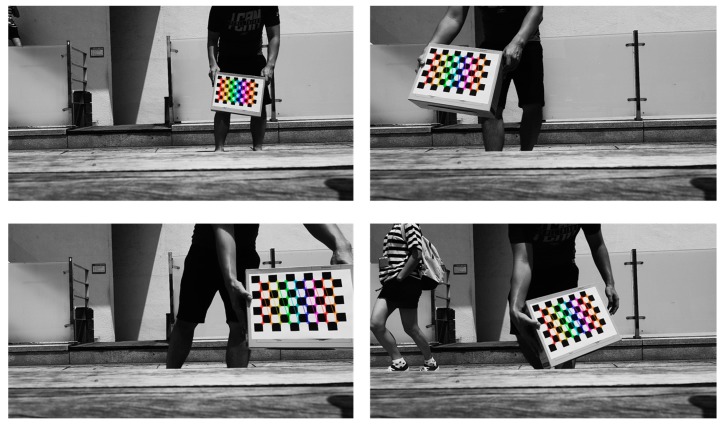
Some examples of our camera calibration process using chessboard pattern.

Before we try to find the pose of our marker which refers to its relative orientation and position with respect to drone camera, we have to perform camera calibration to obtain camera matrix and distortion coefficients vector. As shown in Figure 16, we print out a chessboard pattern image [75] in an A4 paper and take several images with different chessboard’s poses using the DJI Phantom 4 camera. Using OpenCV’s *calibrateCamera* function [76], we obtain camera matrix (*M*) and distortion coefficients (*C*) shown as below:

(14) $$M=\left[\begin{array}{ccc} 749.54 & 0 & 2.5\\ 0 & 1904.2 & 4\\ 0 & 0 & 1 \end{array}\right]$$

(15) $$C=\left[\begin{array}{ccccc} -0.01713 & 6.8e^{-5} & 3.73e^{-4} & 4.62e^{-3} & -6.64e^{-8} \end{array}\right]$$

Our marker pose estimation is carried out through the OpenCV’s *solvePnP* function [76]. The output of this function is the current pose of the camera with respect to the center of the marker. The *solvePnP* function is based on the pinhole camera model. In this model, each point of view is formed by projecting each image point into the corresponding image plane point using a perspective transformation:

(16) sp=A[R|t]P

(17) $$\mathrm{or}\;s\left[\begin{array}{c} u\\ v\\ 1 \end{array}\right]=\left[\begin{array}{ccc} f_{x} & 0 & c_{x}\\ 0 & f_{y} & c_{y}\\ 0 & 0 & 1 \end{array}\right]\left[\begin{array}{cccc} r_{11} & r_{12} & r_{13} & t_{x}\\ r_{21} & r_{22} & r_{23} & t_{y}\\ r_{31} & r_{32} & r_{33} & t_{z} \end{array}\right]\left[\begin{array}{c} X\\ Y\\ Z\\ 1 \end{array}\right]$$

where *X, Y, Z* are coordinates of 3D points *P* in the world coordinates space and *u, v* are the coordinates of the projection point *p* in image plane. The coefficients (*c_x_, c_y_*) and (*f_x_, f_y_*) representing respectively the coordinates of the principal point, that is usually at the image center, and the focal lengths expressed in pixel units. We already mentioned how we obtained these coefficients through the calibration procedure of the camera described above. Using the *solvePnP* function, we can obtain all parameters of rotation matrix (*R*) and translation vector (*t*). In order to get three Euler angles (yaw, pitch, roll) from acquired rotation matrix *R*, we use OpenCV’s *decomposeProjectionMatrix* function [76].

In this experiment, we want to show that our proposed profile checker algorithm is not only capable of detecting marker center or its direction but also support computing full pose of the pattern respect to the camera frame and achieve comparable results with another fiducial marker tracker, such as ArUco tracker [77]. Using the same MUTOH printer [48], we print a predefined version of ArUco marker (DICT_6x6_50) which has the same dimension (1-m width and 1-m height) with our proposed marker. Same camera parameters of Equations (13) and (14) were used for our method and ArUco marker-based method for fair comparison.

Because we cannot have ground-truth values of full pose (yaw, pitch, roll, three translations on X-, Y-, and Z-axes) from drone, we measured the accuracy of full pose estimation by comparing the estimated values of full pose by our method with those by ArUco marker-based method. For that, experiments were done as follows.

**Figure 17:**
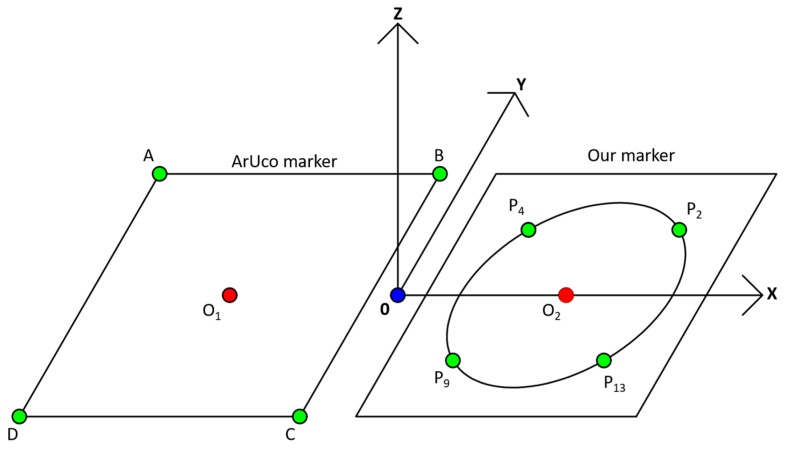
Experimental setup of our marker and ArUco marker for estimating full pose.

Figure 17 shows how we manually place ArUco marker next to our proposed marker, the distance between two marker centers O_1_ and O_2_ is 1.2 m. In detail, the Y- and Z-axes of our marker and ArUco marker are coincident, and the two X-axes of these two markers have only the disparity of 1.2 m. In this case, we let the drone flying freely with lots of X, Y, Z translations and yaw, pitch, roll rotations. Using OpenCV’s *detectMarkers* function [77] which is based on the research [78], we detect 4 corners A, B, C, D of ArUco marker at each image frame and use them as the key points for pose estimation. Applying the same *solvePnP* function described above, we can also compute pose estimation of ArUco marker respected to camera frame. 

In our research, we used our own method for detecting four key points with our marker not using OpenCV function. That is, as shown in Figure 4, we select four detected key points P_2_, P_4_, P_9_, and P_13_ from our proposed profile checker algorithm as 4 key points for pose estimation. The reason we choose these four points is that even if the drone is getting closer to the marker, these four key points would always be seen by the drone’s camera. By conclusion, for our marker, we used our own detection/tracking algorithm for four key points whereas we used their own, freely available detection/tracking code for four key points from ArUco marker [78] for fair comparison. From the four detected key points from our marker and ArUco marker, we used the same OpenCV’s *solvePnP* function [76] in order to obtain the 6 parameters (the 3 translations of X-, Y-, Z-axes, and the 3 rotations of yaw, pitch, roll) for pose estimation. This *solvePnP* function has been widely used for pose estimation purpose in previous researches (even in [78]), and we used the same *solvePnP* function for both our marker and ArUco marker for fair comparisons. Table 6 shows all 3D coordinates of selected key points using for both markers.

**Figure 18:**
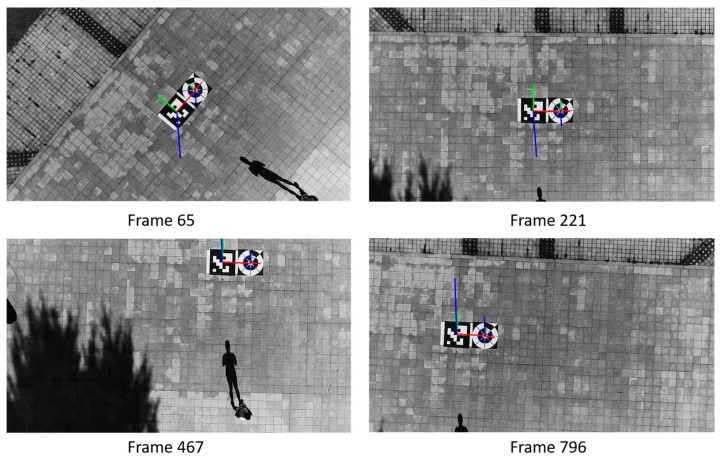
Examples of computed pose estimation of our proposed marker compared to ArUco marker in the case of free style flying.

**Figure 19:**
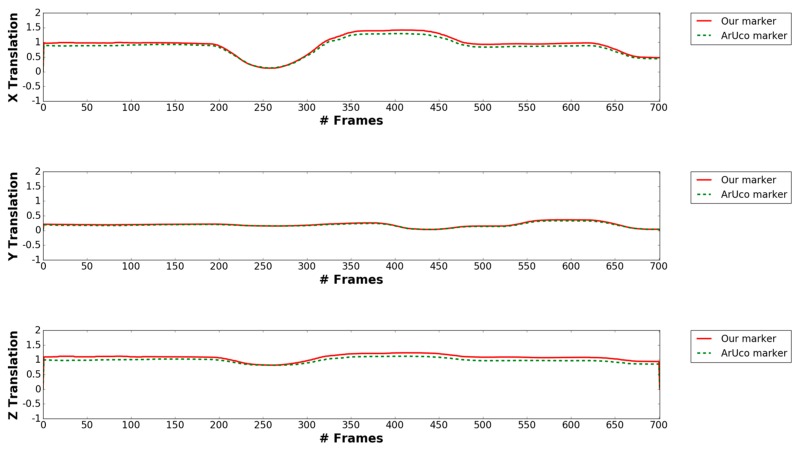
Comparison between estimated X, Y, Z translation of our marker and ArUco marker.

**Figure 20:**
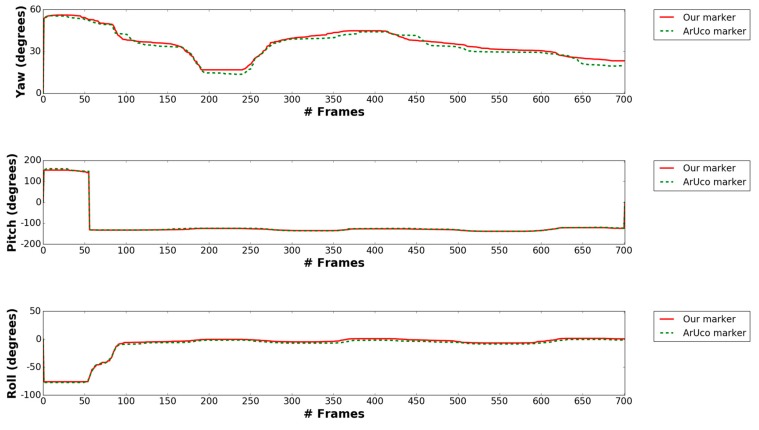
Comparison between estimated yaw, pitch, roll rotations of our marker and ArUco marker.

Figure 18 shows our pose estimation result of our proposed marker and ArUco marker with 3D coordinates axes (red, green and blue line representing X-, Y- and Z-axes). As shown in Figure 18, using key points detected from our proposed method, we successfully achieve comparable results to ArUco marker’s pose estimation. At the 65th, 467th and 796th frames, even though the drone has lot of X, Y, Z translation and rotates a lot in the yaw, pitch and roll, but our marker’s predicted coordinates axes are almost the same with ArUco’s estimation. As shown from Figure 19 and Figure 20, our marker‘s pose estimation (red solid line) is almost the same with ArUco’s pose estimation (green dash line) in all video frames. Table 7 shows average error of X, Y, Z translation and yaw, pitch, roll rotation in the above case. As shown in this table, we can find that the accuracy by our marker-based estimation of full pose is similar to that by ArUco marker-based method.

## 5. Conclusions

In this paper, we proposed a novel method for detecting the marker center and estimating the marker direction based on the ATM, profile checker, and Kalman filtering algorithm in order to precisely land a UAV. In particular, our proposed method can be operated using nighttime video based on the adaptive thresholding and morphological processing algorithm. We performed extensive tests in various environments that show that our algorithm outperformed the state-of-the-art visual trackers in terms of both robustness and accuracy. In addition, the processing speed of our method was much faster than those obtained by previous methods, and we confirmed that our proposed method can be operated at a real-time speed exceeding 40 (1000/25) fps in an actual embedded system.

Based on the specification of DJI phantom 4 drone used in our experiment [36], the maximum wind speed resistance is 10 m/s. However, when we collected lots of data of Table 2 under various weather and time situations, there was no case that the wind speed exceeds in 3.5 m/s, and it is very difficult to collect the data by waiting the weather of strong wind. We would have experiments with the additional data collected at strong wind in future work. Our algorithm can detect the marker center and the estimate marker direction without the need for any training procedures. Therefore, for future work, we hope to enhance the performance of our method by adopting a training scheme, and we will consider employing the deep learning-based tracking algorithm in our system.

## Figures and Tables

**Table 1:** Summary of comparisons of proposed and previous studies.

Categories	Sub-Categories	Type of Camera	Time for Drone Landing	Descriptions	Strength	Weakness
Passive methods		A trinocular system with three visible light FireWire cameras	Daytime [22]	Color landmarks on the UAV are deployed as key features for position estimation by a ground station using CamShift algorithm.	This research not only achieved good results for landing and positioning task, but also practical for real-time applications.	The feature extraction algorithm is not guaranteed to work under low light condition. Complicated set-up of three cameras on the ground is required.
		A pan-tilt unit (PTU) with stereo infrared cameras	Daytime and nighttime with various weather conditions [23]	Several target tracking algorithms have been evaluated and tested on a quadrotor and a fixed-wing aircraft.	It can track target early by using enlarge field of view by PTU. Fast Marching Method is proposed among other techniques by its effiency and accuracy.	Low accuracy in case of fix-wing touchdown points and high temperature objects in the background.
		Two NIR camera array system with NIR laser lamp	Daytime and nighttime with different light conditions [24]	A NIR laser lamp is fixed on the nose of the UAV for easy detection.	A wide baseline camera array-based method was proposed to achieve high precision for calibration and localization results.	It is not practical to be used in the narrow landing area. Complicated set-up of two camera array systems on the ground is required.
Active methods	Without marker	Visible light camera	Daytime and nighttime with guiding lamp [25]	Image binarization and Hough transform used to extract the runway border line for the landing in flight-gear flight simulator.	A simple algorithm is used for border-line detection of runway.	Guiding lamps are required at nighttime, which make it difficult to be used in various places.
			Day time [26]	Two-stage processing procedure to find all possible landing areas and select the best one using the naive Bayesian classifier.	Without the marker, drone can find the landing site in emergency case.	Experiments were not performed in various places and time. In addition, system is evaluated on a Mac Pro laptop computer.
	With marker	Thermal camera	Daytime, and nighttime [38,39]	Using letter-based marker emitting FIR light, feature points can be extracted from the marker so that the drone can perform translation or rotation movements in order to perform safety landing at the desired location.	Using the thermal image, marker detection can be less affected by illumination, time, and environmental change.	A costly thermal camera should be used in drone, and this cannot be used in conventional drone systems, including the use of only visible-light camera.
		Visible light camera	Daytime [24,25,26,27,28,30]	Detecting marker by contour, circle detector or key points descriptor based on SURF, etc.	Marker detection is possible using conventional visible light camera in drone.	Marker is detected only during daytime. High computing algorithm cannot be processed in real-time with on-board system [29]
			Daytime, and nighttime (**proposed method**)	Using real-time marker-based tracking algorithm tested on an onboard system having low processing power.	Marker detection method can be operated both in daytime and nighttime.	A specific marker is required for the proposed method.

**Table 2:** Description of DDroneC-DB1.

Kinds of Sub-Database	Time	Condition	Description
Sub-database 1 (drone landing)	Morning	Humidity: 41.5%, wind speed: 1.4 m/s, temperature: 8.6 °C, spring, sunny, Illuminance:1900 lux	A sunny day with clear sky, which has affected the illumination on the marker Landing speed: 4 m/s Auto mode of camera shutter speed (8~1/8000 s) and ISO (100~3200)
	Afternoon	Humidity: 73.8%, wind speed: 2 m/s, temperature: −2.5 °C, winter, cloudy, Illuminance: 1200 lux	Low level of illumination observed in the winter time, which affected the intensity of background area. Landing speed: 6 m/s Auto mode of camera shutter speed (8~1/8000 s) and ISO (100~3200)
	Evening	Humidity: 38.4%, wind speed: 3.5 m/s, temperature: 3.5 °C, winter, windy, Illuminance: 500 lux	There is the change in the marker’s position due to strong wind Landing speed: 4 m/s Auto mode of camera shutter speed (8~1/8000 s) and ISO (100~3200)
	Night	Humidity: 37.5%, wind speed: 3.2 m/s, temperature: 6.9 °C, spring, foggy, Illuminance: 0.3 lux	Marker cannot be seen owning low level of light at dark night Landing speed: 6 m/s Auto mode of camera shutter speed (8~1/8000 s) and ISO (100~3200)
Sub-database 2 (drone hovering)	Morning	Humidity: 41.6%, wind speed: 2.5 m/s, temperature: 11 °C, spring, foggy, Illuminance: 1000 lux	Drone hovers above the marker, and the marker is manually moved and rotated while capturing videos. Auto mode of camera shutter speed (8~1/8000 s) and ISO (100~3200)
	Afternoon	Humidity: 43.5%, wind speed: 2.8 m/s, temperature: 13 °C, spring, sunny, Illuminance: 1860 lux	
	Evening	Humidity: 42.9%, wind speed: 2.9 m/s, temperature: 10 °C, spring, Illuminance: 600 lux	
	Night	Humidity: 41.5%, wind speed: 3.1 m/s, temperature: 6 °C, spring, dark night, Illuminance: 0.05	

**Table 3:** Comparisons of average CLE obtained our method with those obtained by other methods (pixels).

Categories	Sequence	Ours without KF	Ours with KF	ATM	MIL [40]	TLD [41]	Median Flow [42]	KCF [43]
Sub-database 1	Morning	3.32	3.26	63.98	4.29	103.3	83.45	31.03
	Afternoon	2.91	2.86	15.69	5.58	58.01	92.21	13.86
	Evening	3.89	3.54	28.11	8.85	75.13	95.84	7.42
	Night	8.36	12.2	65.22	28.15	48.1	23.56	31.65
Sub-database 2	Morning	1.98	1.94	38.19	1.92	32.34	6.11	5.58
	Afternoon	2.32	2.05	32.1	5.04	25.66	4.19	3.14
	Evening	1.74	1.68	37.99	7.8	2.98	8.08	1.73
	Night	7.12	9.75	49.78	6.33	15.94	12.37	7.45

**Table 4:** Comparisons of average PDE obtained by our method with those obtained by other methods (degrees).

Categories	Sequence	Ours without KF	Ours with KF	ATM
Sub-database 1	Morning	0.72	0.51	50.94
	Afternoon	0.88	0.47	2.64
	Evening	1.33	1.01	17.37
	Night	4.39	5.9	80.56
Sub-database 2	Morning	2.45	1.97	26.84
	Afternoon	2.79	2.17	43.65
	Evening	2.28	1.83	27.16
	Night	3.35	4.12	68.17

**Table 5:** Comparisons of average processing time achieved by our proposed method with those obtained by other methods (ms).

Categories	Sequence	Ours without KF	Ours with KF	MIL [40]	TLD [41]	Median Flow [42]	KCF [43]
Sub-database 1	Morning	22	23	367	2971	43	359
	Afternoon	22	22	371	2921	44	258
	Evening	22	23	369	2192	43	223
	Night	24	25	740	3993	88	180
Sub-database 2	Morning	20	20	754	3129	92	165
	Afternoon	20	22	768	3427	74	145
	Evening	21	21	762	3419	72	104
	Night	23	25	730	4530	86	151

**Table 6:** 3D coordinates of all detect key points of ArUco marker and our proposed marker.

Methods	Key Points	X	Y	Z
Our marker	P_2_	$0.6+\frac{0.35}{2}$	$\frac{0.35}{2}$	0
	P_4_	$0.6-\frac{0.35}{2}$	$\frac{0.35}{2}$	0
	P_9_	$0.6-\frac{0.35}{2}$	$\frac{-0.35}{2}$	0
	P_13_	$0.6+\frac{0.35}{2}$	$\frac{-0.35}{2}$	0
ArUco marker	A	$-0.6-\frac{1}{2}$	$\frac{1}{2}$	0
	B	$-0.6+\frac{1}{2}$	$\frac{1}{2}$	0
	C	$-0.6+\frac{1}{2}$	$-\frac{1}{2}$	0
	D	$-0.6-\frac{1}{2}$	$-\frac{1}{2}$	0

**Table 7:** Average error of pose estimation in a free flying case scenario.

Category	Average Error between Our Marker and ArUco Marker-Based Methods
X	0.076
Y	0.014
Z	0.095
Yaw	1.8°
Pitch	1.15°
Roll	2.09°

## References

[1] Commercial UAV Market Analysis by Product (Fixed Wing, Rotary Blade, Nano, Hybrid), by Application (Agriculture, Energy, Government, Media & Entertainment) and Segment Forecasts to 2022. http://www.grandviewresearch.com/industry-analysis/commercial-uav-market.

[2] Austin R. (2010). Unmanned Aircraft Systems: Uavs Design, Development and Deployment.

[3] Ham Y., Han K.K., Lin J.J., Golparvar-Fard M. (2016). Visual monitoring of civil infrastructure systems via camera-equipped unmanned aerial vehicles (UAVs): A review of related works. Vis. Eng..

[4] Chan B., Guan H., Jo J., Blumenstein M. (2015). Towards UAV-based bridge inspection systems: A review and an application perspective. Struct. Monit. Maint..

[5] Eschmann C., Kuo C.M., Kuo C.H., Boller C. (2013). High-resolution multisensor infrastructure inspection with unmanned aircraft systems. Int. Arch. Photogramm. Remote Sens. Spat. Inf. Sci..

[6] Máthé K., Buşoniu L. (2015). Vision and control for UAVs: A survey of general methods and of inexpensive platforms for infrastructure inspection. Sensors.

[7] Bryson M., Sukkarieh S., Valavanis K.P., Vachtsevanos G.J. (2015). Inertial sensor–based simultaneous localization and mapping for UAVs. Handbook of Unmanned Aerial Vehicles.

[8] Feldman M.S. (2014). Simultaneous Localization and Mapping Implementations for Navigation of an Autonomous Robot. Bachelor’s Thesis.

[9] Dehghan S.M.M., Moradi H. (2016). SLAM–inspired simultaneous localization of UAV and RF sources with unknown transmitted power. Trans. Inst. Meas. Control.

[10] Cruz G.C.S., Encarnação P.M.M. (2012). Obstacle avoidance for unmanned aerial vehicles. J. Intell. Robot. Syst..

[11] Gageik N., Benz P., Montenegro S. (2015). Obstacle detection and collision avoidance for a UAV with complementary low–cost sensors. IEEE Access..

[12] Call B.R. (2006). Obstacle Avoidance for Small Unmanned Air Vehicles. Master’s Thesis.

[13] Barry A.J. (2016). High–Speed Autonomous Obstacle Avoidance with Pushbroom Stereo. Ph.D. Thesis.

[14] Gottlieb Y., Shima T. (2015). UAVs task and motion planning in the presence of obstacles and prioritized targets. Sensors.

[15] Partsinevelos P., Agadakos I., Athanasiou V., Papaefstathiou I., Mertikas S., Kyritsis S., Tripolitsiotis A., Zervos P. On–board computational efficiency in real time UAV embedded terrain reconstruction. Proceedings of the the European Geosciences Union General Assembly.

[16] Bulatov D., Solbrig P., Gross H., Wernerus P., Repasi E., Heipke C. (2011). Context–based urban terrain reconstruction from UAV–videos for geoinformation applications. Int. Arch. Photogramm. Remote Sens. Spat. Inform. Sci..

[17] Witayangkurn A., Nagai M., Honda K., Dailey M., Shibasaki R. (2011). Real–time monitoring system using unmanned aerial vehicle integrated with sensor observation service. Int. Arch. Photogramm. Remote Sens. Spat. Inform. Sci..

[18] Nagai M., Witayangkurn A., Honda K., Shibasaki R. (2012). UAV–based sensor web monitoring system. Int. J. Navig. Observ..

[19] Baiocchi V., Dominici D., Milone M.V., Mormile M. (2013). Development of a software to plan UAVs stereoscopic flight: An application on post earthquake scenario in L’Aquila city. Lect. Notes Comput. Sci..

[20] Yeong S.P., King L.M., Dol S.S. (2015). A review on marine search and rescue operations using unmanned aerial vehicles. Int. J. Mech. Aerosp. Ind. Mech. Manuf. Eng..

[21] Amazon Prime Air. https://www.amazon.com/Amazon–Prime–Air/b?node=8037720011.

[22] Martínez C., Campoy P., Mondragón I., Olivares–Méndez M.A. Trinocular ground system to control UAVs. Proceedings of the IEEE/RSJ International Conference on Intelligent Robots and Systems.

[23] Kong W., Zhang D., Wang X., Xian Z., Zhang J. Autonomous landing of an UAV with a ground–based actuated infrared stereo vision system. Proceedings of the IEEE/RSJ International Conference on Intelligent Robots and Systems.

[24] Yang T., Li G., Li J., Zhang Y., Zhang X., Zhang Z., Li Z. (2016). A ground–based near infrared camera array system for UAV auto–landing in GPS–denied environment. Sensors.

[25] Anitha G., Kumar R.N.G. (2012). Vision based autonomous landing of an unmanned aerial vehicle. Procedia Eng..

[26] Li X. (2013). A software scheme for UAV’s safe landing area discovery. AASRI Procedia.

[27] Sharp C.S., Shakernia O., Sastry S.S. A vision system for landing an unmanned aerial vehicle. Proceedings of the the IEEE International Conference on Robotics and Automation.

[28] Lange S., Sünderhauf N., Protzel P. Autonomous landing for a multirotor UAV using vision. Proceedings of the the International Conference on Simulation, Modeling and Programming for Autonomous Robots.

[29] Zhao Y., Pei H. (2012). An improved vision–based algorithm for unmanned aerial vehicles autonomous landing. Phys. Procedia.

[30] Chaves S.M., Wolcott R.W., Eustice R.M. (2015). NEEC research: Toward GPS–denied landing of unmanned aerial vehicles on ships at sea. Nav. Eng. J..

[31] Ling K. (2014). Precision Landing of a Quadrotor UAV on a Moving Target Using Low–Cost Sensors. Master’s Thesis.

[32] AprilTag. https://april.eecs.umich.edu/software/apriltag.html.

[33] Kyristsis S., Antonopoulos A., Chanialakis T., Stefanakis E., Linardos C., Tripolitsiotis A., Partsinevelos P. (2016). Towards autonomous modular UAV missions: The detection, geo–location and landing paradigm. Sensors.

[34] AprilTags C++ Library. http://people.csail.mit.edu/kaess/apriltags/.

[35] Linux for Tegra R27.1. https://developer.nvidia.com/embedded/linux–tegra.

[36] DJI. http://www.dji.com.

[37] Jetson TK1. http://www.nvidia.com/object/jetson–tk1–embedded–dev–kit.html.

[38] Xu G., Zhang Y., Ji S., Cheng Y., Tian Y. (2009). Research on computer vision–based for UAV autonomous landing on a ship. Pattern Recognit. Lett..

[39] Xu G., Qi X., Zeng Q., Tian Y., Guo R., Wang B. (2013). Use of land’s cooperative object to estimate UAV’s pose for autonomous landing. Chin. J. Aeronaut..

[40] Babenko B., Yang M.H., Belongie S. Visual tracking with online multiple instance learning. Proceedings of the IEEE Conference on Computer Vision and Pattern Recognition.

[41] Kalal Z., Mikolajczyk K., Matas J. (2012). Tracking–learning–detection. IEEE Trans. Pattern Anal. Mach. Intell..

[42] Kalal Z., Mikolajczyk K., Matas J. Forward–backward error: Automatic detection of tracking failures. Proceedings of the International Conference on Pattern Recognition.

[43] Henriques J.F., Caseiro R., Martins P., Batista J. (2015). High–speed tracking with kernelized correlation filters. IEEE Trans. Pattern Anal. Mach. Intell..

[44] Bay H., Ess A., Tuytelaars T., Gool L.V. (2006). Speeded–up robust features (SURF). Comput. Vis. Image Underst..

[45] Lowe D.G. (2004). Distinctive image features from scale–invariant keypoints. Int. J. Comput. Vis..

[46] Calonder M., Lepetit V., Strecha C., Fua P. Brief: Binary robust independent elementary features. Proceedings of the European Conference on Computer Vision.

[47] Rublee E., Rabaud V., Konolige K., Bradski G. ORB: An efficient alternative to SIFT or SURF. Proceedings of the IEEE International Conference on Computer Vision.

[48] MUTOH Printer. https://www.mutoh.eu/.

[49] Lam S.K., Yeong C.Y., Yew C.T., Chai W.S., Suandi S.A. (2010). A study on similarity computations in template matching technique for identity verification. Int. J. Comput. Sci. Eng..

[50] Li H., Duan H.B., Zhang X.Y. (2010). A novel image template matching based on particle filtering optimization. Pattern Recognit. Lett..

[51] Türkan M., Guillemot C. Image prediction: Template matching vs. sparse approximation. Proceedings of the 17th International Conference on Image Processing.

[52] Lin Y., Chunbo X. Template matching algorithm based on edge detection. Proceedings of the International Symposium on Computer Science and Society.

[53] Lu X., Shi Z. (2010). Detection and tracking control for air moving target based on dynamic template matching. J. Electron. Meas. Instrum..

[54] Paravati G., Esposito S. (2014). Relevance–based template matching for tracking targets in FLIR imagery. Sensors.

[55] Opromolla R., Fasano G., Rufino G., Grassi M. (2015). A model–based 3D template matching technique for pose acquisition of an uncooperative space object. Sensors.

[56] Welch G., Bishop G. An introduction to the Kalman filter. Proceedings of the Special Interest Group on GRAPHics and Interactive Techniques (SIGGRAPH).

[57] Zaitoun N.M., Aqel M.J. (2015). Survey on image segmentation techniques. Procedia Comput. Sci..

[58] Salman N. (2006). Image segmentation based on watershed and edge detection techniques. Int. Arab. J. Inf. Technol..

[59] Belaid L.J., Mourou W. (2009). Image segmentation: A watershed transformation algorithm. Image Anal. Stereol..

[60] Bala A. (2012). An improved watershed image segmentation technique using MATLAB. Int. J. Sci. Eng. Res..

[61] Yahya A.A., Tan J., Hu M. (2013). A novel model of image segmentation based on watershed algorithm. Adv. Multimed..

[62] Uyun S., Hartati S., Harjoko A., Choridah L. (2015). A comparative study of thresholding algorithms on breast area and fibroglandular tissue. Int. J. Adv. Comput. Sci. Appl..

[63] Gonzalez R.C., Woods R.E. (2010). Digital Image Processing.

[64] ARM Processors. https://www.arm.com/products/processors.

[65] Lee H., Jung S., Shim D.H. Vision–based UAV landing on the moving vehicle. Proceedings of the International Conference on Unmanned Aircraft System.

[66] Fu C., Duan R., Kircali D., Kayacan E. (2016). Onboard robust visual tracking for UAVs using a reliable global–local object model. Sensors.

[67] Intel® NUC Kit NUC5i7RYH. https://ark.intel.com/products/87570/Intel–NUC–Kit–NUC5i7RYH.

[68] OpenCV 3.1. http://opencv.org/opencv–3–1.html.

[69] Microsoft Visual Studio. https://www.visualstudio.com/.

[70] CMake. https://cmake.org/.

[71] Stanford Drone Dataset. http://cvgl.stanford.edu/projects/uav_data/.

[72] Mini–Drone Video Dataset. http://mmspg.epfl.ch/mini–drone.

[73] SenseFly Dataset. https://www.sensefly.com/drones/example–datasets.html.

[74] Dongguk Drone Camera Database (DDroneC–DB1). http://dm.dgu.edu/link.html.

[75] Chessboard Pattern. http://docs.opencv.org/3.1.0/pattern.png.

[76] Camera Calibration and 3D Reconstruction. http://docs.opencv.org/2.4/modules/calib3d/doc/camera_calibration_and_3d_reconstruction.html.

[77] Detection of ArUco Markers. http://docs.opencv.org/trunk/d5/dae/tutorial_aruco_detection.html.

[78] Garrido-Jurado S., Muñoz-Salinas R., Madrid-Cuevas F.J., Marín-Jiménez M.J. (2014). Automatic generation and detection of highly reliable fiducial markers under occlusion. Pattern Recognit..

